# Invasive versus non-invasive management of older patients with non-ST elevation myocardial infarction (SENIOR-NSTEMI): a cohort study based on routine clinical data

**DOI:** 10.1016/S0140-6736(20)30930-2

**Published:** 2020-08-29

**Authors:** Amit Kaura, Jonathan A C Sterne, Adam Trickey, Sam Abbott, Abdulrahim Mulla, Benjamin Glampson, Vasileios Panoulas, Jim Davies, Kerrie Woods, Joe Omigie, Anoop D Shah, Keith M Channon, Jonathan N Weber, Mark R Thursz, Paul Elliott, Harry Hemingway, Bryan Williams, Folkert W Asselbergs, Michael O'Sullivan, Graham M Lord, Narbeh Melikian, Thomas Johnson, Darrel P Francis, Ajay M Shah, Divaka Perera, Rajesh Kharbanda, Riyaz S Patel, Jamil Mayet

**Affiliations:** a National Institute for Health Research Imperial Biomedical Research Centre, Imperial College London and Imperial College Healthcare NHS Trust, London, UK; b National Institute for Health Research Bristol Biomedical Research Centre, University of Bristol and University Hospitals Bristol NHS Foundation Trust, Bristol, UK; c Department of Population Health Sciences, University of Bristol, Bristol, UK; d National Institute for Health Research Oxford Biomedical Research Centre, University of Oxford and Oxford University Hospitals NHS Foundation Trust, Oxford, UK; e National Institute for Health Research King's Biomedical Research Centre, King's College London, Guy's and St Thomas' NHS Foundation Trust and King's College Hospital NHS Foundation Trust, London, UK; f National Institute for Health Research University College London Biomedical Research Centre, University College London and University College London Hospitals NHS Foundation Trust, London, UK; g Institute of Health Informatics, Health Data Research UK, London, UK; h National Institute for Health Research Cambridge Biomedical Research Centre, University of Cambridge and Cambridge University Hospitals NHS Foundation Trust, Cambridge, UK; i National Institute for Health Research Manchester Biomedical Research Centre, University of Manchester and Manchester University NHS Foundation Trust, Manchester, UK

## Abstract

**Background:**

Previous trials suggest lower long-term risk of mortality after invasive rather than non-invasive management of patients with non-ST elevation myocardial infarction (NSTEMI), but the trials excluded very elderly patients. We aimed to estimate the effect of invasive versus non-invasive management within 3 days of peak troponin concentration on the survival of patients aged 80 years or older with NSTEMI.

**Methods:**

Routine clinical data for this study were obtained from five collaborating hospitals hosting NIHR Biomedical Research Centres in the UK (all tertiary centres with emergency departments). Eligible patients were 80 years old or older when they underwent troponin measurements and were diagnosed with NSTEMI between 2010 (2008 for University College Hospital) and 2017. Propensity scores (patients' estimated probability of receiving invasive management) based on pretreatment variables were derived using logistic regression; patients with high probabilities of non-invasive or invasive management were excluded. Patients who died within 3 days of peak troponin concentration without receiving invasive management were assigned to the invasive or non-invasive management groups based on their propensity scores, to mitigate immortal time bias. We estimated mortality hazard ratios comparing invasive with non-invasive management, and compared the rate of hospital admissions for heart failure.

**Findings:**

Of the 1976 patients with NSTEMI, 101 died within 3 days of their peak troponin concentration and 375 were excluded because of extreme propensity scores. The remaining 1500 patients had a median age of 86 (IQR 82–89) years of whom (845 [56%] received non-invasive management. During median follow-up of 3·0 (IQR 1·2–4·8) years, 613 (41%) patients died. The adjusted cumulative 5-year mortality was 36% in the invasive management group and 55% in the non-invasive management group (adjusted hazard ratio 0·68, 95% CI 0·55–0·84). Invasive management was associated with lower incidence of hospital admissions for heart failure (adjusted rate ratio compared with non-invasive management 0·67, 95% CI 0·48–0·93).

**Interpretation:**

The survival advantage of invasive compared with non-invasive management appears to extend to patients with NSTEMI who are aged 80 years or older.

**Funding:**

NIHR Imperial Biomedical Research Centre, as part of the NIHR Health Informatics Collaborative.

## Introduction

Most patients with a non-ST elevation myocardial infarction (NSTEMI) are aged 70 years or older.^1^ The proportion of the global population aged 80 years or older is projected to triple over the next 20 years.^2^ Increasing age is a key predictor of adverse events in patients with coronary artery disease: older patients presenting with an acute coronary syndrome are at higher risk of short-term and long-term adverse outcomes compared with younger patients.^3^, ^4^ However, the rate of invasive coronary angiography declines with age. Only 38% of patients with NSTEMI who are aged 81 years or older receive a coronary angiogram, compared with 78% of those aged 60 years or younger.^5^

Large randomised trials showed a long-term survival advantage for invasive management compared with non-invasive management of NSTEMI, but the mean age of participants was 66 years. Few patients in their 80s were enrolled into these studies and the survival benefit cannot be assumed to translate to these patients.^6^ Because of the perceived higher risks of invasive procedures, many physicians manage only a minority of older patients with NSTEMI invasively.

**Research in context**

**Evidence before this study**

Electronic searches of MEDLINE, EMBASE, and the Cochrane Central Register of Controlled Trials were done for articles in English published from inception of these databases to Jan 20, 2019. Studies with the following characteristics were identified: (1) randomised trials or observational studies, either restricted to populations aged 75 years or older reporting outcomes for this patient subgroup; (2) patients presenting with non-ST elevation myocardial infarction (NSTEMI); and (3) comparison of invasive management (coronary angiography or percutaneous coronary intervention or coronary artery bypass grafting during index admission) with non-invasive management during the index hospital admission. Only 38% of patients with NSTEMI aged 81 years or older receive a coronary angiogram, compared with 78% of those aged 60 years or younger. In routine care, frail patients with multiple comorbidities are less likely to be treated invasively. Large randomised trials have shown a long-term survival advantage for invasive compared with non-invasive management of NSTEMI, but the mean age of these participants was 66 years. Few patients aged 80 years or older were enrolled into these studies and as a result the survival benefit cannot be assumed to translate to older patients. Because the evidence to support invasive management is not conclusive, many physicians treat older patients with NSTEMI symptomatically, offering invasive management only to selected patients, such as those with ongoing chest pain.The European Society of Cardiology and American Heart Association guidelines suggest that older patients with NSTEMI should be considered for invasive management with coronary angiography and revascularisation, but there is conflicting evidence for this recommendation. Only two small randomised trials and two post-hoc subgroup analyses of larger randomised trials have evaluated invasive versus non-invasive management for NSTEMI in older patients: a meta-analysis that pooled data from these trials did not find clear evidence that invasive therapy reduced long-term mortality (odds ratio 0·84, 95% CI 0·66–1·06).Although registry studies have attempted to address whether the benefit of invasive management extends to older patients, their results might have been affected by immortal time bias, because patients who died early in the course of their presentation, before invasive therapy could be considered or arranged, were assigned to the non-invasive group.

**Added value of this study**

To our knowledge this is the first study to estimate the effect of invasive compared with non-invasive management of NSTEMI on the survival of patients aged 80 years or older using multicentre, routine clinical data and methods that help to minimise bias in analyses of observational data by considering the target trial that would answer the clinical question. This study provides evidence that the survival advantage from invasive management might extend to patients aged 80 years or older with NSTEMI (adjusted hazard ratio 0·68, 95% CI 0·55–0·84).

**Implications of all the available evidence**

This study strengthens the evidence for an invasive approach to management of patients aged 80 years or older with NSTEMI. Ideally, clinical decision making should be driven by findings from randomised trials. In the absence of randomised trial data, clinical decisions need to be made on the basis of the best available evidence. An invasive approach might be the better management strategy in patients who could be managed either invasively or non-invasively.

Registry studies have attempted to address whether the benefit of invasive management extends to older patients.^7^, ^8^, ^9^ In routine care, frail patients with multiple comorbidities are much more likely to be treated non-invasively whereas the fittest patients are much more likely to undergo invasive management. Although these studies attempted to control for this confounding by indication, their results might have been affected by immortal time bias^10^, ^11^ because patients who died early in the course of their presentation—before invasive therapy could be considered or arranged—were assigned to the non-invasive group.

Questions about comparative effectiveness should ideally be answered using randomised trials.^12^ The SENIOR-RITA trial^13^ aims to determine whether an invasive compared with a non-invasive management strategy reduces time to cardiovascular death or non-fatal myocardial infarction in patients aged 75 years or older with NSTEMI. However, the final completion date is not expected until 2029.^13^

Estimates of comparative effectiveness from observational databases might be improved through specification of the hypothetical (target) randomised trial, the results of which would answer the specific clinical question of interest.^14^ We estimated the effect of invasive management compared with non-invasive management on survival in patients aged 80 years or older with NSTEMI using multicentre routinely collected clinical data (SENIOR-NSTEMI study), using methods that help to minimise bias in analyses of observational data through consideration of the target trial.

## Methods

### Study design and participants

The National Institute for Health Research (NIHR) Health Informatics Collaborative was established to facilitate the sharing and routine reuse of clinical data for translational research.^15^, ^16^ Data for this study were obtained from five collaborating hospitals hosting NIHR Biomedical Research Centres (Imperial College Healthcare, London; University College Hospital, London; Oxford University Hospitals, Oxford; King's College Hospital, London; and Guys & St Thomas' Hospital, London) in the UK, which were all tertiary centres with emergency departments.

We defined the target trial as comparing patients who had invasive management for NSTEMI (defined as coronary angiography with or without subsequent revascularisation within 3 days of peak troponin concentration) with patients who did not receive such invasive management. We followed all included patients from the time of their peak troponin concentration until death or censoring in April, 2017.

Eligible patients were 80 years old or older when they underwent troponin measurements and were diagnosed with NSTEMI between 2010 (2008 for University College Hospital) and 2017. Only the first episode of hospital care with troponin measurements was eligible. Classification of NSTEMI was made on the basis of the assigned International Statistical Classification of Diseases and Related Health Problems discharge codes (I21.4; acute subendocardial myocardial infarction).^17^ Patients with a concurrent primary diagnosis of an acute illness associated with possible oxygen supply-and-demand mismatch were excluded.

We used the target trial to minimise bias by starting follow up at the time of peak troponin; ensuring that early deaths did not influence the definition of the intervention group, to avoid immortal time bias; excluding patients with a high probability of being assigned to one of the treatment groups (patients included in a trial must be eligible to receive each treatment); using regression models that included the propensity score to control confounding; and doing intention-to-treat analyses, in which patients who received invasive intervention after the first three days were analysed in the non-invasive management group. This study was approved by the London-South East Research Ethics Committee (REC reference: 16/HRA/3327).

### Outcomes

The primary outcome was all-cause mortality. The secondary outcome was the number of hospital admissions for heart failure during follow-up. In post-hoc analyses we examined new hospital admissions for bleeding, stroke, acute coronary syndrome, and further invasive management. Bleeding was defined as minor or major bleeding. Vital status was ascertained using the national Patient Demographic Service, which incorporates national death registry information and local notifications. We could only ascertain heart failure admission using electronic health record data in the hospital where their initial NSTEMI diagnosis was made.

### Statistical analysis

In a randomised trial, follow-up starts at the time that treatment strategies are assigned. In the absence of such assignment, follow-up in patients who do not undergo invasive management is consistent with both treatment strategies for the first 3 days. Classification of patients who die within 3 days of peak troponin concentration without invasive management to the comparison group can cause an immortal time bias because some patients could have had invasive management had they not died.^10^, ^11^ To limit such bias, patients who died within 3 days of peak troponin concentration were excluded from the initial modelling steps, then included separately.

Propensity scores (patients' estimated probability of receiving invasive management) were derived using logistic regression, for patients who did not die within 3 days of peak troponin concentration. The final propensity score model was based on backwards stepwise selection, with a p value threshold of 0·2. We considered the following pretreatment variable groups: patient demographics, blood test results, cardiovascular risk factors, cardiovascular disease, renal disease, respiratory disease, neurological disease, psychiatric disease, other comorbidities, and markers of frailty. The Comorbidity Domain of the Frailty Index was calculated as a score ranging between 0 and 1.^18^ All the variables considered and those selected into the propensity score model are listed in the appendix (pp 9–10). Non-linear relationships were modelled using smoothing splines. Analyses used the first measurement of each haematological and biochemical blood test during the hospital care episode, except for troponin concentration for which the peak measurement was used.

We examined the number of patients and deaths in each treatment group, within strata defined by propensity score quantiles. Participants in a randomised trial must be eligible to receive any of the interventions being compared. Therefore, patients in a stratum for which there were few patients or deaths in either group were excluded, to make it more likely that analyses were restricted to patients eligible to receive either treatment strategy. In such patients, inverse probability of treatment weights were defined as 1/propensity score in patients who received invasive management and as 1/(1–propensity score) in patients who received non-invasive management.

We used Kaplan-Meier plots to display the cumulative risk of mortality and hospital admission for heart failure over time in each treatment group. The plots were weighted for inverse probability of treatment to estimate the cumulative risk had all eligible patients been assigned to one or the other group.

We used Cox models (stratified by centre) to estimate mortality hazard ratios (HR) comparing invasive management with non-invasive management in patients who did not die within 3 days of their peak troponin concentration and who were in the eligible propensity score strata. Two approaches were used to control confounding: multivariable adjustment (including the propensity score modelled using a restricted cubic spline) and inverse probability of treatment weighting. The variables adjusted for were chosen using backwards stepwise selection, with a p value threshold of 0·2, and treatment group was included in all models. The same variables were considered for inclusion in the Cox models as for the propensity score model (appendix pp 9–10). We then included patients in eligible propensity score strata who died within 3 days of their peak troponin concentration. Those who had received invasive intervention were classified in the invasive management group: the others (who might have received invasive intervention had they not died) were randomly assigned to the invasive or non-invasive management group on the basis of their propensity scores. We generated 20 datasets, estimated mortality HRs in each, then pooled the results using Rubin's rules to estimate the overall HR. The pretreatment variables included in these Cox model analyses are listed in the appendix (pp 9–10).

In sensitivity analyses, we quantified the potential for unmeasured confounding to explain the effect of invasive management on estimated mortality hazard ratios by calculating the E value.^19^ We investigated the effect of assigning all patients who died within 3 days of their peak troponin concentration to one or the other treatment group and of reducing the number of eligible propensity score strata. Changes in the HR with age were investigated using cubic splines. The number of hospital admissions relating to heart failure in the invasive management and non-invasive management groups were compared using negative binomial regression adjusted by centre. The pretreatment variables included were selected using backwards stepwise selection, with a p value threshold of 0·2, and are listed in the appendix (pp 9–10).

Statistical analyses used R (version 3.5.0) and Stata (version 16.0). The data acquisition plan is in the appendix (pp 19–23). The study is registered with ClinicalTrials.gov, NCT03507309.

### Role of the funding source

The funder had no role in study design, data collection, data analysis, data interpretation, or writing the report. AK, JACS, and AT had access to all data and take full responsibility for its integrity and the accuracy of the analyses. The corresponding author had final responsibility for the decision to submit for publication.

## Results

Of the 61 342 patients aged 80 years or older who had troponin measured in the study period Jan, 2010 (Jan, 2008 for University College Hospital, London, UK) to April, 2017, 2788 (4·5%) had a diagnosis of NSTEMI. 116 (4·2%) patients were excluded because of missing data in one or more variables and 696 (25%) had a concurrent primary diagnosis with an acute illness (appendix p 11).

**Figure 1:**
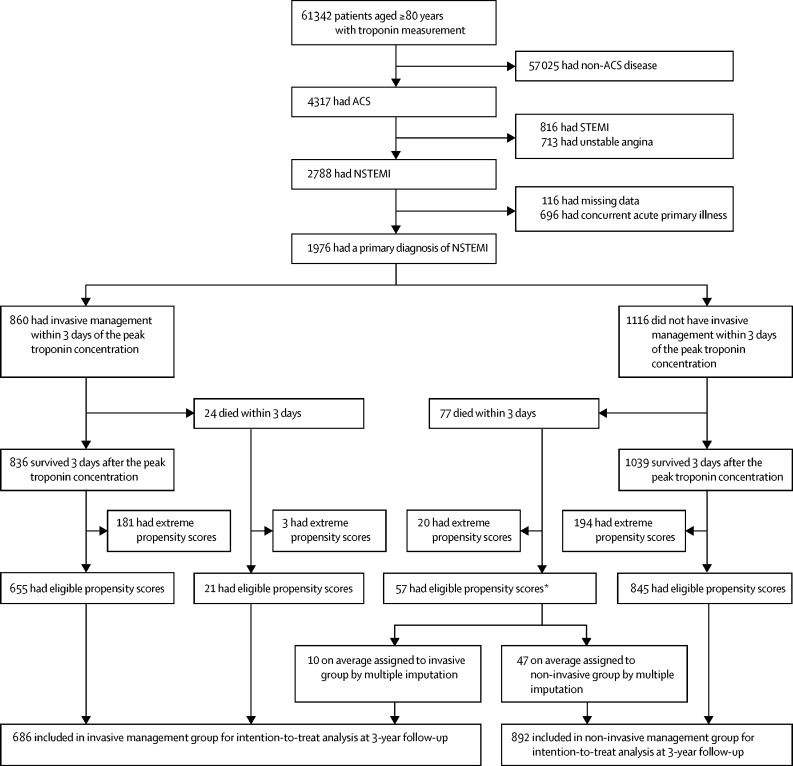
Study profile ACS=acute coronary syndrome. NSTEMI=non-ST elevation myocardial infarction. Invasive management defined as angiography with or without revascularisation within 3 days of peak troponin concentration. *57 of the 77 deaths in patients not invasively managed within 3 days of the peak troponin concentration had eligible propensity scores and were assigned to treatment groups using multiple imputation. Across the imputed datasets, on average, ten were included in the invasive management group and 47 were included in the non-invasive management group.

Of the 1976 patients included, 961 (49%) underwent invasive management during their index admission of whom 860 (89%) had invasive management during the first 3 days after peak troponin concentration (appendix p 3). There were 890 (45%) deaths in the 1976 patients included, of which 101 were within the first 3 days after peak troponin concentration. These 101 patients were excluded from the next stage of the analyses (figure 1). The characteristics of these patients are shown in the appendix (pp 12–13).

**Figure 2:**
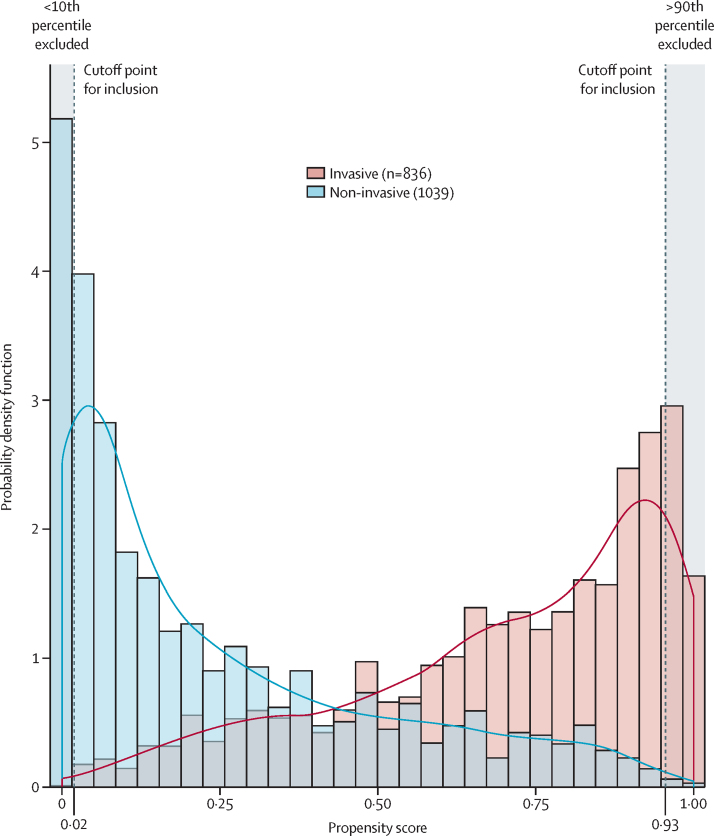
Combined histogram and probability density function of the propensity score for 1875 patients with non-ST elevation myocardial infarction in patients aged 80 years or older according to invasive or non-invasive management strategy Invasive management defined as angiography with or without revascularisation within 3 days of peak troponin concentration. Patients with propensity scores <10% had a high probability of receiving non-invasive treatment and those with a score >90% had a high probability of receiving invasive treatment; these patients were excluded from analyses.

Characteristics of the patients that were associated with receiving invasive management are shown in the appendix (pp 4–5, 14–15). The propensity score distributions for patients treated invasively and non-invasively are shown in figure 2**.** The proportion of patients who died during follow-up in each group, according to propensity score quantiles, are shown in table 1. In both treatment groups, the proportion who died was higher in patients with lower propensity scores (those most likely to be non-invasively managed). Below the tenth percentile (propensity score=0·0178) none of the 188 patients were invasively managed and above the 90th percentile (propensity score=0·9292) only six (3%) of 187 patients were non-invasively managed. Therefore, analyses were restricted to patients whose propensity scores were between the tenth and 90th percentiles (figure 1). The characteristics of patients very likely to receive non-invasive interventions (<10th percentile) and those very likely to receive invasive management (>90th percentile) are shown in the appendix (pp 12–13).

**Table 1:** Proportion of deaths in the invasive management and non-invasive management groups, according to percentiles of the propensity score for the study population who survived up to 3 days from peak troponin concentration

	**Propensity score upper limit**	**Invasive management group (n=836)**		**Non-invasive management group (n=1039)**		**Hazard ratio (95% CI)**
		Number of patients	Number of deaths (%)	Number of patients	Number of deaths (%)	
99 to 100	1·0000	19	3 (16%)	0	0	..
95 to <99	0·9936	73	20 (27%)	2	0	..
90 to <95	0·9633	89	23 (26%)	4	0	..
75 to <90	0·9292	232	53 (23%)	49	14 (29%)	0·64 (0·34–1·18)
50 to <75	0·7742	294	85 (29%)	176	69 (39%)	0·61 (0·44–0·85)
25 to <50	0·4154	117	41 (35%)	352	177 (50%)	0·49 (0·35–0·69)
10 to <25	0·0987	12	6 (50%)	268	168 (63%)	0·72 (0·32–1·63)
5 to <10	0·0178	0	0	95	62 (65%)	..
1 to <5	0·0054	0	0	74	52 (70%)	..
0 to <1	0·0007	0	0	19	16 (84%)	..
Overall	..	836	231 (28%)	1039	558 (54%)	0·34 (0·29–0·40)

Invasive management defined as an invasive procedure within 3 days of peak troponin concentration.

The 1500 patients included in the next stage of analyses had a median age of 86 (IQR 82–89) years (table 2; appendix pp 12–13). Revascularisation was done in 486 (74%) of 655 patients undergoing invasive management. The strongest predictors of invasive management were interhospital transfer (odds ratio [OR] 3·19, 95% CI 2·08–4·89) and family history of ischaemic heart disease (2·84, 2·01–3·99), whereas interstitial lung disease (0·22, 0·09–0·58) and indicators of frailty were strong predictors of non-invasive management (table 2).

**Table 2:** Characteristics of participants who survived for at least 3 days after peak troponin concentration according to invasive or non-invasive management strategy

		**Invasive management (n=655)**	**Non-invasive management (n=845)**	**Mean difference (95% CI)**	**Odds ratio (95% CI)**
**Numerical characteristics**					
Demographic characteristics					
	Age (years)	85·3 (4·3)	86·9 (4·9)	1·6 (1·2 to 2·1)	..
Haematology and biochemistry results					
	C-reactive protein (mg/L)	25·2 (45·2)	35·8 (56·2)	10·6 (5·4 to 15·9)	..
	Creatinine (μmol/L)	113·7 (85·9)	121·5 (94·9)	7·8 (−1·5 to 17·1)	..
	Haemoglobin (g/dL)	12·5 (1·9)	12·1 (1·9)	−0·3 (−0·5 to −0·2)	..
	Platelet count (10^9^ cells per L)	229·1 (74·9)	236·3 (82·7)	7·1 (−1·0 to 15·2)	..
	Potassium (mmol/L)	4·3 (0·6)	4·3 (0·6)	0·1 (−0·0 to 0·1)	..
	Sodium (mmol/L)	136·9 (4·2)	137·1 (4·9)	0·2 (−0·2 to 0·7)	..
	Troponin (upper limit of normal)	270·2 (510·4)	177·6 (375·7)	−93 (−137 to −48)	..
	White cell count (10^9^ cells per L)	9·6 (3·5)	10·1 (4·0)	0·5 (0·1 to 0·9)	..
Frailty					
Comorbidity domain of Frailty Index score		0·22 (0·09)	0·23 (0·09)	0·01 (0·00 to 0·02)	..
**Binary characteristics**					
Demographic characteristics					
	Interhospital transfer	73 (11%)	32 (4%)	..	3·19 (2·08 to 4·89)
	Male sex	390 (60%)	418 (50%)	..	1·50 (1·22 to 1·85)
Cardiovascular risk factors					
	Diabetes	172 (26%)	201 (24%)	..	1·14 (0·90 to 1·44)
	Family history of ischaemic heart disease	108 (16%)	55 (7%)	..	2·84 (2·01 to 3·99)
	Hypercholesterolaemia	275 (42%)	253 (30%)	..	1·69 (1·37 to 2·10)
	Hypertension	406 (62%)	456 (54%)	..	1·39 (1·13 to 1·71)
	Tobacco use	237 (36%)	152 (18%)	..	2·59 (2·04 to 3·28)
Cardiovascular disease					
	Abdominal aortic aneurysm	3 (1%)	7 (1%)	..	0·55 (0·14 to 2·14)
	Angina	132 (20%)	160 (19%)	..	1·08 (0·84 to 1·40)
	Aortic stenosis	34 (5%)	57 (7%)	..	0·76 (0·49 to 1·17)
	Atrial fibrillation	105 (16%)	155 (18%)	..	0·85 (0·65 to 1·12)
	Cardiogenic shock	6 (1%)	7 (1%)	..	1·11 (0·37 to 3·31)
	Cardiac arrest	10 (2%)	13 (2%)	..	0·99 (0·43 to 2·28)
	Complete heart block	9 (1%)	14 (2%)	..	0·83 (0·36 to 1·92)
	Heart failure	110 (17%)	205 (24%)	..	0·63 (0·49 to 0·82)
	Peripheral vascular disease	40 (6%)	46 (5%)	..	1·13 (0·73 to 1·75)
	Previous myocardial infarction	498 (76%)	475 (56%)	..	2·47 (1·97 to 3·09)
	Supraventricular tachycardia	1 (0·2%)	3 (0·4%)	..	0·43 (0·04 to 4·14)
	Ventricular fibrillation	5 (0·8%)	7 (1%)	..	0·92 (0·29 to 2·91)
	Ventricular tachycardia	9 (1%)	8 (1%)	..	1·46 (0·56 to 3·80)
Renal disease					
	Acute renal failure	33 (5%)	74 (9%)	..	0·55 (0·36 to 0·84)
	Chronic kidney disease (>stage 2)	48 (7%)	70 (8%)	..	0·88 (0·60 to 1·28)
	Urinary tract infection	17 (3%)	60 (7%)	..	0·35 (0·20 to 0·60)
Respiratory disease					
	Interstitial lung disease	5 (0·8%)	28 (3%)	..	0·22 (0·09 to 0·58)
	Obstructive lung disease	84 (13%)	117 (14%)	..	0·92 (0·68 to 1·24)
	Other lung disease	65 (10%)	116 (14%)	..	0·69 (0·50 to 0·96)
	Pneumonia	35 (5%)	78 (9%)	..	0·56 (0·37 to 0·84)
	Pulmonary embolism	3 (0·5%)	2 (0·2%)	..	1·94 (0·32 to 11·6)
	Respiratory failure	9 (1%)	25 (3%)	..	0·46 (0·21 to 0·99)
Neurological disease					
	Ischaemic stroke	6 (0·9%)	17 (2%)	..	0·45 (0·18 to 1·15)
	Parkinson's disease	5 (0·8%)	7 (1%)	..	0·92 (0·29 to 2·91)
	Subdural haemorrhage	2 (0·3%)	1 (0·1%)	..	2·58 (0·23 to 28·6)
Psychiatric disease					
	Alcohol misuse	20 (3%)	44 (5%)	..	0·57 (0·33 to 0·98)
	Anxiety	4 (0·6%)	15 (2%)	..	0·34 (0·11 to 1·03)
	Bipolar disease	1 (0·2%)	4 (0·4%)	..	0·32 (0·04 to 2·88)
	Delirium	3 (0·5%)	15 (2%)	..	0·25 (0·07 to 0·88)
	Depression	9 (1%)	18 (2%)	..	0·64 (0·29 to 1·43)
	Other psychiatric illness	59 (9%)	91 (11%)	..	0·82 (0·58 to 1·16)
Other comorbidities					
	Arthritis	13 (2%)	37 (4%)	..	0·44 (0·23 to 0·84)
	Constipation	14 (2%)	23 (3%)	..	0·78 (0·40 to 1·53)
	Fracture	5 (0·8%)	12 (1%)	..	0·53 (0·19 to 1·52)
	Gastric ulcer	2 (0·3%)	4 (0·4%)	..	0·64 (0·12 to 3·53)
	Haemorrhage	25 (4%)	29 (3%)	..	1·12 (0·65 to 1·93)
	Inflammatory disorder	57 (9%)	114 (13%)	..	0·61 (0·44 to 0·86)
	Malignancy	43 (7%)	81 (10%)	..	0·66 (0·45 to 0·97)
	Metabolic disorder	5 (0·8%)	23 (3%)	..	0·27 (0·10 to 0·73)
	Sepsis	6 (0·9%)	22 (3%)	..	0·35 (0·14 to 0·86)
Frailty					
	Bowel incontinence	11 (2%)	36 (4%)	..	0·38 (0·19 to 0·76)
	Dementia	9 (1%)	41 (5%)	..	0·27 (0·13 to 0·57)
	History of falls	16 (2%)	63 (7%)	..	0·31 (0·18 to 0·54)
	Impaired hearing	6 (0·9%)	22 (3%)	..	0·35 (0·14 to 0·86)
	Impaired vision	3 (0·5%)	15 (2%)	..	0·25 (0·07 to 0·88)
	Mild cognitive impairment (no dementia)	5 (0·8%)	19 (2%)	..	0·33 (0·12 to 0·90)
	Need for assistance at home	57 (9%)	113 (13%)	..	0·62 (0·44 to 0·86)
	Need for mobility assistance	41 (6%)	84 (10%)	..	0·60 (0·41 to 0·89)
	Need for personal care assistance	6 (0·9%)	22 (3%)	..	0·35 (0·14 to 0·86)
	Reduced mobility	9 (1%)	26 (3%)	..	0·44 (0·20 to 0·94)
	Speaking difficulty	6 (0·9%)	22 (3%)	..	0·35 (0·14 to 0·86)
	Urinary catheterisation	5 (0·8%)	11 (1%)	..	0·58 (0·20 to 1·69)
	Urinary incontinence	14 (2%)	18 (2%)	..	1·00 (0·50 to 2·03)
	Weight loss	7 (1%)	23 (3%)	..	0·39 (0·16 to 0·91)

All data are mean (SD) for numerical characteristics or n (%) for binary characteristics. Only participants who survived for 3 days after peak troponin concentration were included. Comparisons between groups are unadjusted and are quantified as mean differences for numerical characteristics and odds ratios for binary characteristics.

**Figure 3:**
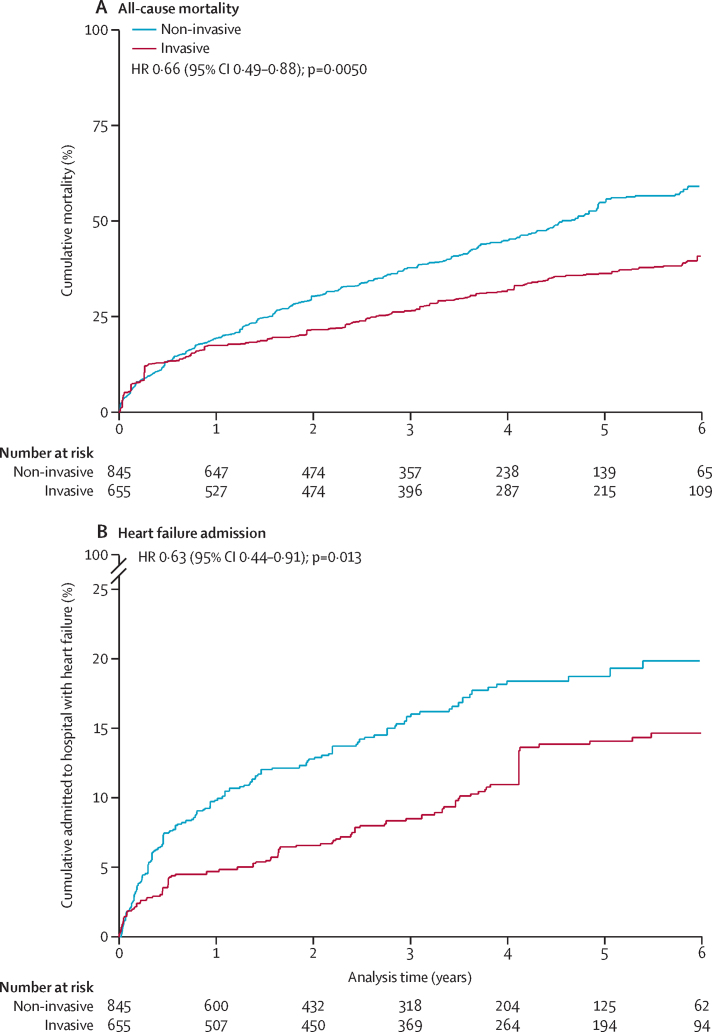
Kaplan-Meier curves displaying cumulative all-cause mortality and probability of admission for heart failure according to invasive and non-invasive management (A) Cumulative all-cause mortality. (B) Probability of admission for heart failure. Plots are weighted according to the inverse probability of treatment received. They compare outcomes if all eligible patients were invasively or non-invasively managed. Patient deaths within 3 days of peak troponin were excluded. Hazard ratios are inverse probability of treatment weighted, excluding deaths within 3 days.

During median 3·0 (IQR 1·2–4·8) years follow-up there were 613 (41%) deaths. At 5 years, Kaplan-Meier estimates of cumulative mortality from 3 days after peak troponin concentration were 31% (168 events) in the invasive management group and 61% (413 events) in the non-invasive group (appendix p 6). Inverse probability of treatment weighted Kaplan-Meier plots show an estimated 5-year cumulative mortality of 36% if all these patients received invasive intervention, compared with 55% if all received non-invasive intervention (figure 3A).

The crude mortality HR comparing invasive with non-invasive management from 3 days after peak troponin concentration was 0·38 (95% CI 0·32–0·46), attenuated to 0·56 (0·45–0·70) after multivariable adjustment for clinical characteristics and propensity score. The mortality HR was further attenuated (0·66, 0·49–0·88] following inverse probability of treatment weighting (table 3).

**Table 3:** Estimated mortality hazard ratios comparing invasive with non-invasive management, for patients with non-ST elevation myocardial infarction aged 80 years or older

	**Number in the invasive group**	**Number in the non-invasive group**	**Hazard ratio (95% CI)**	**p value**
**Primary analysis**				
Multivariable* plus PS adjustment, with deaths within 3 days of peak troponin concentration assigned to a treatment group based on PS†	686	892	0·68 (0·55–0·84)	<0·0001
**Sensitivity analyses**				
Crude, excluding deaths within 3 days of peak troponin concentration	655	845	0·38 (0·32–0·46)	<0·0001
Multivariable* plus PS adjustment, excluding deaths within 3 days	655	845	0·56 (0·45–0·70)	<0·0001
IPTW, excluding deaths within 3 days	655	845	0·66 (0·49–0·88)	0·0050
Multivariable* plus PS adjustment, restricted to patients with PS in the 25th and 75th percentiles, excluding deaths within 3 days	411	528	0·56 (0·44–0·71)	<0·0001
Multivariable* plus PS adjustment, with deaths within 3 days of peak troponin concentration assigned to the non-invasive group	655	923	0·50 (0·40–0·61)	<0·0001
Multivariable* plus PS adjustment, with deaths within 3 days of peak troponin concentration assigned to the invasive group	733	845	1·05 (0·86–1·27)	0·65

IPTW=inverse probability of treatment weighted. PS=propensity score.

* Adjusted for age, interhospital transfer, creatinine and haemoglobin concentration, family history of ischaemic heart disease, hypercholesterolaemia, hypertension, abdominal aortic aneurysm, angina, aortic stenosis, cardiogenic shock, heart failure, previous myocardial infarction, supraventricular tachycardia, ventricular fibrillation, ventricular tachycardia, acute renal failure, urinary tract infection, interstitial lung disease, obstructive lung disease, other lung disease, ischaemic stroke, Parkinson's disease, anxiety, gastric ulcer, metabolic disorder, bowel incontinence, dementia, history of falls, mild cognitive impairment (no dementia), need for mobility assistance, and speaking difficulty.

† 57 of the 77 deaths in patients not invasively managed within 3 days of the peak troponin concentration had eligible propensity scores and were assigned to treatment groups using multiple imputation. Across the imputed datasets, on average, ten were included in the invasive management group and 47 were included in the non-invasive management group.

The propensity score distributions for the 101 patients who died within 3 days of their peak troponin concentration are shown in the appendix (p 7). 77 (76%) patients received non-invasive management and 78 patients had a propensity score between the 10th and 89th percentiles. When these patients were assigned to treatment groups based on their propensity score using multiple imputation (primary analysis), the mortality HR was 0·68 (95% CI 0·55–0·84). The E value for unmeasured confounding was 1·94. The adjusted HRs were 1·05 (0·86–1·27) after assigning all patients who died within 3 days of their peak troponin concentration to the invasive group and 0·50 (0·40–0·61) after assigning them to the non-invasive group (table 3).

Including patients whose propensity score were between the 25th and 75th percentiles (figure 2) led to an adjusted HR of 0·56 (95% CI 0·44–0·71) from 3 days after peak troponin concentration. There was no evidence of an association between invasive management and lower mortality during the first month of follow-up, although the strongest associations of invasive management with lower mortality were from 1 year of follow-up onwards (appendix p 16). The association of invasive management with lower mortality appeared to attenuate with increasing age, but smaller patient numbers at the oldest ages meant that the confidence intervals on age-specific HRs were wide (appendix p 8).

The numbers of patients who were invasively managed during follow-up were 103 (16%) of 655 in the invasive management group and 161 (19%) of 845 in the non-invasive group (appendix p 17). 120 (14%) of 845 patients in the non-invasive group were invasively managed between 4 and 28 days after their peak troponin. However, the proportion of patients who were invasively managed after 28 days was smaller for the non-invasive group than in the invasive group. Invasive management was done during a recurrent acute coronary syndrome admission in only 43 (5%) patients in the invasive group and 71 (8%) patients in the non-invasive group. Of those undergoing further invasive management in the invasive group, 68 (66%) had revascularisation, compared with 24 (15%) of those in the non-invasive management group during their index hospital admission.

The number of patients admitted with heart failure during follow up was 76 (mean 0·20 admissions; range 0–7) in the invasive group and 127 (mean 0·24 admissions; range 0–6) in the non-invasive group. At 5 years, the cumulative percentages admitted to hospital with heart failure were 15% in the invasive group and 22% in the non-invasive group (appendix p 6). An inverse probability of treatment weighted Kaplan-Meier plot estimated 5-year cumulative admission rates to be 14% if all eligible patients were invasively managed and 19% if all eligible patients were non-invasively managed (figure 3B). Invasive management was associated with a lower incidence of hospital admissions for heart failure than non-invasive management (adjusted incidence rate ratio 0·67, 95% CI 0·48–0·93; appendix p 18). Association between invasive management and additional causes of hospital admission are reported in the appendix (pp 2, 18).

## Discussion

We used multicentre routine clinical data from the NIHR Health Informatics Collaborative to estimate the effect of invasive management compared with non-invasive management on survival and other outcomes in patients with NSTEMI aged 80 years or older. We used a framework for comparative effectiveness research based on explicit description of the target trial whose results would answer the clinical question.^14^ The estimated 32% lower mortality in patients who receive invasive management compared with those who receive non-invasive management strengthens the evidence for an invasive approach. Invasive management was associated with a lower incidence of heart failure hospitalisations, acute coronary syndrome, and invasive management during follow-up.

Patients aged 80 years or older represent a growing proportion of the population presenting with NSTEMI, but these patients are much less likely to receive invasive management. Data from the National Inpatient Sample database in the USA showed that coronary angiography was done in 78% of patients with NSTEMI aged 60 years or younger, compared with 38% of patients aged 81 years or older.^5^ In our study, 49% of eligible patients underwent invasive management during their index admission. These differences do not necessarily reflect ageism, rather they reflect insufficient data to guide clinical practice. Treatment decisions are often made in the context of careful evaluation of potential risks and benefits, estimated life expectancy, and comorbidities. In our study, patients managed non-invasively had worse prognosis and were more likely to have a history of heart failure, chronic kidney disease, malignancy, and lung disease. Patients managed invasively were more likely to have higher troponin concentrations, blood test results within the normal range, and cardiovascular risk factors (including smoking, family history of ischaemic heart disease, hypertension, and hypercholesterolaemia). Such risk factors are generally associated with better prognosis in patients who have had an index event, such as myocardial infarction.^20^, ^21^

The European Society of Cardiology guidelines suggest that older patients should be considered for invasive management and revascularisation (class 2a recommendation: “conflicting evidence and/or a divergence of opinion about the usefulness/efficacy of the given treatment or procedure” but the “weight of evidence/opinion is in favour of usesfulness/efficacy”.^22^ The American Heart Association have a similar guideline recommendation.^23^ Only two small randomised trials (After Eighty^24^ [invasive group n=229 and non-invasive group n=228] and Italian Elderly ACS Trial^25^ [invasive group n=154 and non-invasive group n=159]) and two small post-hoc subgroup analyses of randomised trials (TACTICS-TIMI 18^26^ and FIR^27^) have evaluated invasive management versus non-invasive management for NSTEMI in patients aged 75–80 years or older. A meta-analysis that pooled these data, published in 2017, did not find clear evidence that invasive management reduced long-term mortality (OR 0·84, 95% CI 0·66–1·06; p=0·15).^28^ Interpretation is difficult because TACTICS-TIMI 18 compared an early invasive with a selective invasive strategy with predischarge ischaemia test, following which 49% of patients underwent coronary catheterisation and 32% had revascularisation.^26^ Similarly, the FIR trials, which reported cardiovascular mortality but not all-cause mortality, compared an invasive strategy with a selective invasive strategy, which involved a predischarge ischaemia test, following which nearly half of patients had revascularisation during follow-up.^27^ In the Italian Elderly ACS Study, 29% of patients randomly assigned to receive non-invasive management had coronary catheterisation.^25^ In our study, 161 (19%) of 845 patients analysed in the non-invasive group underwent invasive management during follow-up, of whom only 24 (15%) were revascularised. There were inconsistencies between the four trials with regard to the timing of angiography after randomisation, ranging from 4 h to 7 days, and the duration of patient follow-up following randomisation, ranging from 6 months to 5 years.^24^, ^25^, ^26^, ^27^

The ongoing SENIOR-RITA trial^13^ aims to randomly assign 2300 patients with NSTEMI aged 75 years or older to receive invasive or non-invasive management. With a 5-year follow-up planned, the primary outcome is a composite of cardiovascular death and non-fatal myocardial infarction, with an estimated study completion in 2029.^13^

In the absence of compelling evidence from randomised trials, a small number of registry studies^7^, ^8^, ^9^ have suggested a benefit from invasive therapy, but the findings might have been exaggerated by immortal time bias and the inclusion of very frail patients who were almost certain to be managed non-invasively. Immortal time bias can occur when patients who would have received active intervention are analysed in the no intervention or conservative intervention group because they died before active intervention was implemented.^10^, ^11^ Studies of temporal trends using registry data from the USA and Europe suggest that over the past 15 years the progressive switch from a non-invasive to more invasive approach in older patients with NSTEMI has been accompanied by declining mortality.^5^, ^29^, ^30^

We analysed a large, detailed clinical dataset assembled from routinely collected data on unselected patients. All-cause mortality is the outcome of most interest to patients. However, we could not identify deaths that were related to cardiac pathology. In older patients, deaths from causes, such as cancer, that are not affected by management of NSTEMI are common. This suggests that any effect of invasive management on cardiovascular mortality is likely to be greater than its effect on all-cause mortality. We were unable to explore some outcomes of interest (eg, independent living and quality of life). The five centres contributing data were all tertiary centres with most patients being admitted directly. Only 105 (7%) of 1500 patients of the cohort were transferred from other hospitals to these centres. Therefore, we believe that the findings are generalisable to other hospitals which directly admit older patients with NSTEMI.

The main limitations of our study relate to its observational nature: we cannot exclude bias due to uncontrolled (residual) confounding. However, we took several steps to minimise bias, using specification of a target trial to guide our analyses. We assigned patients who died within 3 days of their peak troponin concentration to the two treatment groups according to their propensity scores, unless they had been invasively managed before death. 3 days was chosen as the optimal time to capture most of the patients who underwent invasive management in our cohort (appendix p 3) and the median time used in previous randomised trials.^24^, ^25^ Patients who received invasive management after 3 days were analysed in the non-invasive group, as would be the case in an intention-to-treat analysis of a randomised trial.

Confounding by indication is a major concern for interpretation of our results: patients with worse prognosis were markedly more likely to receive non-invasive management. We considered more than 70 potentially confounding variables, including patient demographics, comorbidities, and cardiovascular risk factors. We excluded patients in the propensity score strata in which there were few patients or deaths in either group and controlled for potential confounding by a large range of prognostic factors. However, our results could be biased by unmeasured confounding, by factors such as frailty that might not be well captured in routinely collected data. We did not have information on whether there was differential receipt of evidence-based cardiac care in the non-invasive management group, including prescription of medications. Additionally, we did not have data on postadmission prognostic factors that might have affected choice of invasive or non-invasive management within the first 3 days. Calculation of the E value showed that to explain the effect of invasive management on mortality estimated in our study, an unmeasured confounder would need at minimum to have a risk ratio of 1·94 with both undergoing invasive management and mortality, having controlled for all the measured confounders.^19^

Our study strengthens the evidence for an invasive approach to management of older patients with NSTEMI. Ideally, clinical decision making should be driven by randomised trials. However, the only ongoing trial designed to answer this question is not due for completion until the end of the current decade.^13^ In the meantime, clinical decisions need to be made based on the best available evidence, which suggests that an invasive approach might be the better strategy for older patients who could be managed either invasively or non-invasively. Further research into the threshold (based on prognostic factors and comorbidities) at which invasive management should be considered would further assist clinical decision making.

On the basis of routinely collected clinical data from five UK tertiary centres we found that invasive management in patients aged 80 years or older with NSTEMI was associated with 32% lower mortality, compared with non-invasive management. The survival advantage bestowed by invasive management might extend to patients aged 80 years or older with NSTEMI.

## Data sharing

The datasets generated or analysed, or both, during this study are not publicly available because of governance restrictions.

## References

[1] Myocardial Ischaemia National Audit Project (2018). How the NHS cares for patients with heart attack. Annual Public Report April 2016–March 2017.

[2] UN (2019). World Population Prospects 2019: Highlights.

[3] Avezum A, Makdisse M, Spencer F (2005). Impact of age on management and outcome of acute coronary syndrome: observations from the Global Registry of Acute Coronary Events (GRACE). Am Heart J.

[4] Granger CB, Goldberg RJ, Dabbous O (2003). Predictors of hospital mortality in the global registry of acute coronary events. Arch Intern Med.

[5] Rashid M, Fischman DL, Gulati M (2019). Temporal trends and inequalities in coronary angiography utilization in the management of non-ST-Elevation acute coronary syndromes in the U.S. Sci Rep.

[6] Sinclair H, Kunadian V (2016). Coronary revascularisation in older patients with non-ST elevation acute coronary syndromes. Heart.

[7] Gierlotka M, Gąsior M, Tajstra M (2013). Outcomes of invasive treatment in very elderly Polish patients with non-ST-segment-elevation myocardial infarction from 2003–2009 (from the PL-ACS registry). Cardiol J.

[8] Bauer T, Koeth O, Jünger C (2007). Effect of an invasive strategy on in-hospital outcome in elderly patients with non-ST-elevation myocardial infarction. Eur Heart J.

[9] Devlin G, Gore JM, Elliott J, GRACE Investigators (2008). Management and 6-month outcomes in elderly and very elderly patients with high-risk non-ST-elevation acute coronary syndromes: The Global Registry of Acute Coronary Events. Eur Heart J.

[10] Suissa S (2008). Immortal time bias in pharmaco-epidemiology. Am J Epidemiol.

[11] Giobbie-Hurder A, Gelber RD, Regan MM (2013). Challenges of guarantee-time bias. J Clin Oncol.

[12] Luce BR, Kramer JM, Goodman SN (2009). Rethinking randomized clinical trials for comparative effectiveness research: the need for transformational change. Ann Intern Med.

[13] http://ClinicalTrials.gov.

[14] Hernán MA, Robins JM (2016). Using Big Data to emulate a target trial when a randomized trial is not available. Am J Epidemiol.

[15] Kaura A, Panoulas V, Glampson B (2019). Association of troponin level and age with mortality in 250 000 patients: cohort study across five UK acute care centres. BMJ.

[16] Kaura A, Arnold AD, Panoulas V (2020). Prognostic significance of troponin level in 3121 patients presenting with atrial fibrillation (The NIHR Health Informatics Collaborative TROP-AF study). J Am Heart Assoc.

[17] WHO (2016). International statistical classification of diseases and related health problems 10th Revision. https://icd.who.int/browse10/2016/en.

[18] Ritt M, Ritt JI, Sieber CC, Gaßmann KG (2017). Comparing the predictive accuracy of frailty, comorbidity, and disability for mortality: a 1-year follow-up in patients hospitalized in geriatric wards. Clin Interv Aging.

[19] VanderWeele TJ, Ding P (2017). Sensitivity analysis in observational research: introducing the E-value. Ann Intern Med.

[20] Hernán MA, Hernández-Díaz S, Robins JM (2004). A structural approach to selection bias. Epidemiology.

[21] Dahabreh IJ, Kent DM (2011). Index event bias as an explanation for the paradoxes of recurrence risk research. JAMA.

[22] Roffi M, Patrono C, Collet JP, ESC Scientific Document Group (2016). 2015 ESC Guidelines for the management of acute coronary syndromes in patients presenting without persistent ST-segment elevation: Task Force for the Management of Acute Coronary Syndromes in Patients Presenting without Persistent ST-Segment Elevation of the European Society of Cardiology (ESC). Eur Heart J.

[23] Amsterdam EA, Wenger NK, Brindis RG (2014). 2014 AHA/ACC guideline for the management of patients with non-st-elevation acute coronary syndromes: a report of the American College of Cardiology/American Heart Association Task Force on Practice Guidelines. J Am Coll Cardiol.

[24] Tegn N, Abdelnoor M, Aaberge L (2016). Invasive versus conservative strategy in patients aged 80 years or older with non-ST-elevation myocardial infarction or unstable angina pectoris (After Eighty study): an open-label randomised controlled trial. Lancet.

[25] Savonitto S, Cavallini C, Petronio AS (2012). Early aggressive versus initially conservative treatment in elderly patients with non-ST-segment elevation acute coronary syndrome: a randomized controlled trial. JACC Cardiovasc Interv.

[26] Bach RG, Cannon CP, Weintraub WS (2004). The effect of routine, early invasive management on outcome for elderly patients with non-ST-segment elevation acute coronary syndromes. Ann Intern Med.

[27] Damman P, Clayton T, Wallentin L (2012). Effects of age on long-term outcomes after a routine invasive or selective invasive strategy in patients presenting with non-ST segment elevation acute coronary syndromes: a collaborative analysis of individual data from the FRISC II - ICTUS - RITA-3 (FIR) trials. Heart.

[28] Gnanenthiran SR, Kritharides L, D'Souza M (2017). Revascularisation compared with initial medical therapy for non-ST-elevation acute coronary syndromes in the elderly: a meta-analysis. Heart.

[29] Elbadawi A, Elgendy IY, Ha LD (2019). National trends and outcomes of percutaneous coronary intervention in patients ≥70 years of age with acute coronary syndrome (from the National Inpatient Sample Database). Am J Cardiol.

[30] Schoenenberger AW, Radovanovic D, Windecker S, Iglesias JF, Pedrazzini G, Stuck AE, Erne P, AMIS Plus Investigators (2016). Temporal trends in the treatment and outcomes of elderly patients with acute coronary syndrome. Eur Heart J.

