# Supplementary material for: Invasive versus non-invasive management of older patients with non-ST elevation myocardial infarction (SENIOR-NSTEMI): a cohort study based on routine clinical data
Source: Lancet. 2020 Aug 29;396(10251):623–34. doi: 10.1016/S0140-6736(20)30930-2 (PMC7456783; doi:10.1016/S0140-6736(20)30930-2)
Supplement: Supplementary appendix [file mmc1.pdf]

THE LANCET

Supplementary appendix

This appendix formed part of the original submission and has been peer reviewed. We post it as supplied by the authors.

Supplement to: Kaura A, Sterne JAC, Trickey A, et al. Invasive versus non-invasive management of older patients with non-ST elevation myocardial infarction (SENIOR-NSTEMI): a cohort study based on routine clinical data. *Lancet* 2020; **396:** 623–34.

Supplementary Material

Invasive versus non-invasive management of elderly patients with non-ST elevation myocardial infarction(SENIOR-NSTEMI): a cohort study based on routine clinical data

Amit Kaura, Jonathan A C Sterne, Adam Trickey, Sam Abbott, Abdulrahim Mulla, Benjamin Glampson, Vasileios Panoulas, Jim Davies, Kerrie Woods, Joe Omigie, Anoop D Shah, Keith M Channon, Jonathan N Weber, Mark R Thursz, Paul Elliott, Harry Hemingway, Bryan Williams, Folkert W Asselbergs, Michael O'Sullivan, Graham M Lord, Narbeh Melikian, Thomas Johnson, Darrel P Francis, Ajay M Shah, Divaka Perera, Rajesh Kharbanda, Riyaz S Patel, Jamil Mayet

Post-hoc analysis and discussion……………………………………………………………………2
Supplementary Figures..………………………………………………………………………………3–8
Supplementary Tables…………………………………………………………………………………9–18
Data acquisition plan…………………………………………………………………………………19–23

Post-hoc Analysis and Discussion

Associations of invasive management with additional causes of hospital admission.

References are to the literature cited in the main text

Methods

Hazard ratios for first hospital admission for bleeding, stroke, acute coronary syndrome, and further invasive management were estimated using Cox models with baseline hazard stratified by centre. The pre-treatment variables included were selected using backwards stepwise selection with p value threshold 0.2 and are listed in Supplementary Table S1.

Results

Supplementary Table S7 shows that there was little evidence that rates of admission for bleeding (adjusted HR 0.93, 0.52-1.65) or for stroke (1.44, 0.61-3.40) differed between the intervention groups. Rates of hospital admission for acute coronary syndrome appeared lower in invasively managed patients (0.67, 0.45-1.00). Rates of invasive management during follow-up were markedly lower in invasively managed patients (adjusted HR 0.46, 95% CI 0.34-0.61).

Discussion

Consistent with pooled data from previous RCTs[28], we found that rates of hospital admission for acute coronary syndrome and of invasive management during follow-up were lower in patients undergoing invasive than non-invasive management during their index hospital admission. We found no evidence that rates of admission due to bleeding differed between intervention groups. While earlier RCTs found an increased risk of bleeding at long-term follow-up in elderly patients with NSTEMI who were managed invasively[26], the more recent After Eighty Trial[24] did not confirm this. This may reflect the increased use of radial access during angiography (90% in the After Eighty Trial[24]) and a reduction in the use of antiplatelet medications such as glycoprotein IIIb/IIIa inhibitors that are associated with excess bleeding in older populations.

Supplementary Figure S1. Histogram showing the distribution of the number of days from peak troponin level to invasive management, among 935 patients who were invasively managed during their admission (49% of 1976 eligible patients).

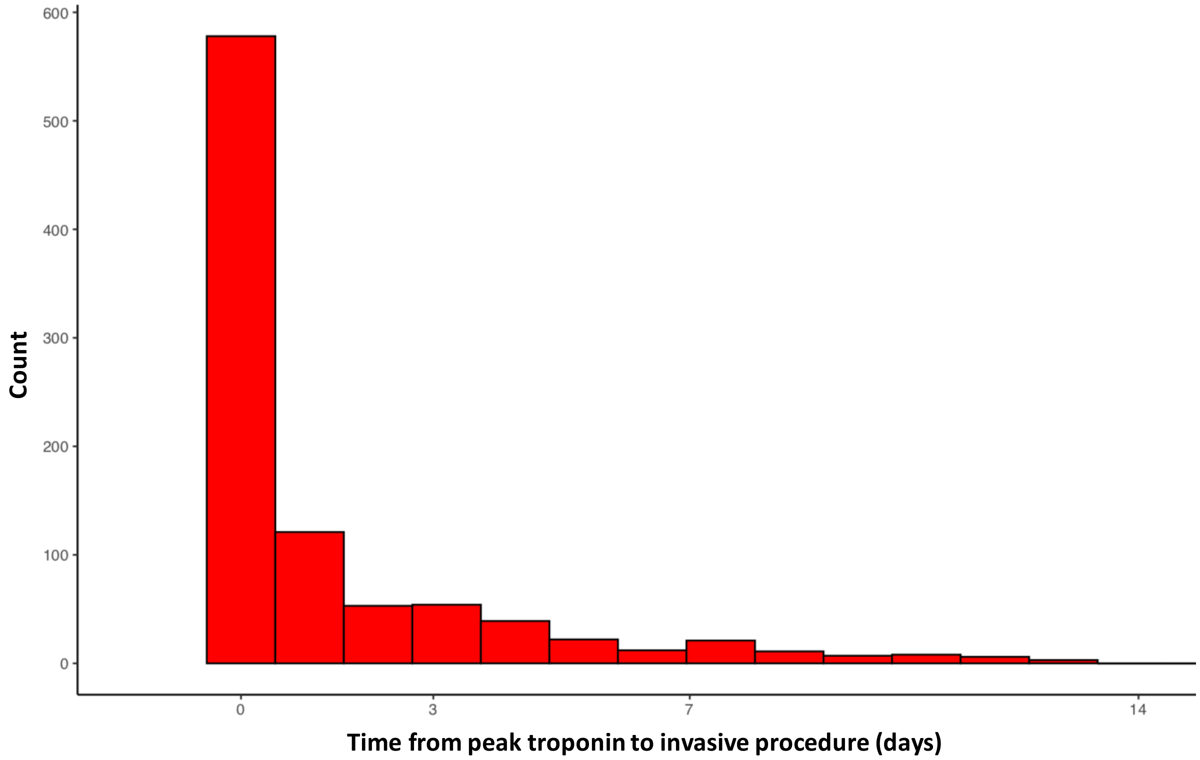

Supplementary Figure S2. **Associations of numerical patient characteristics with invasive management, modelled using smoothing splines**

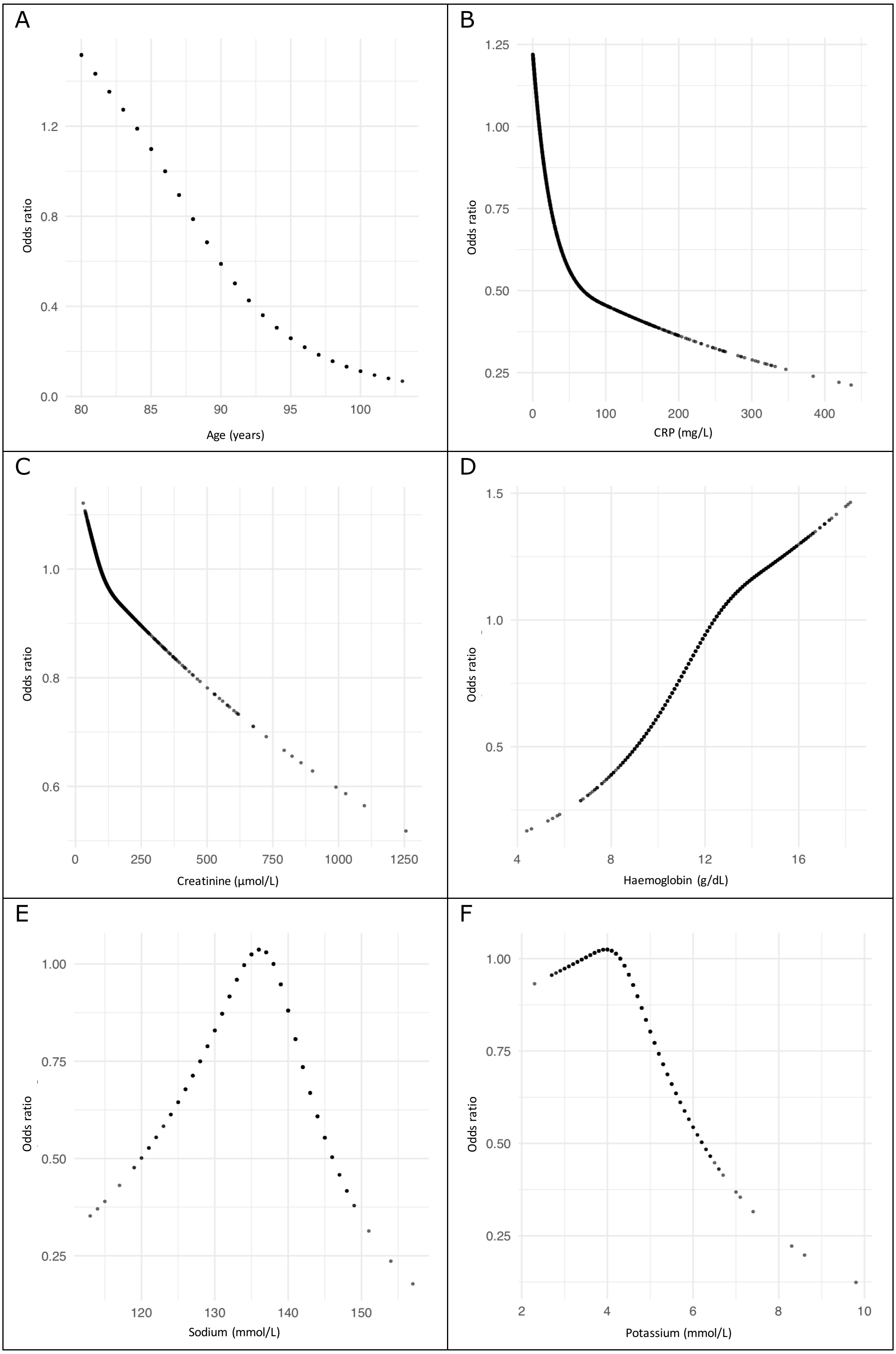

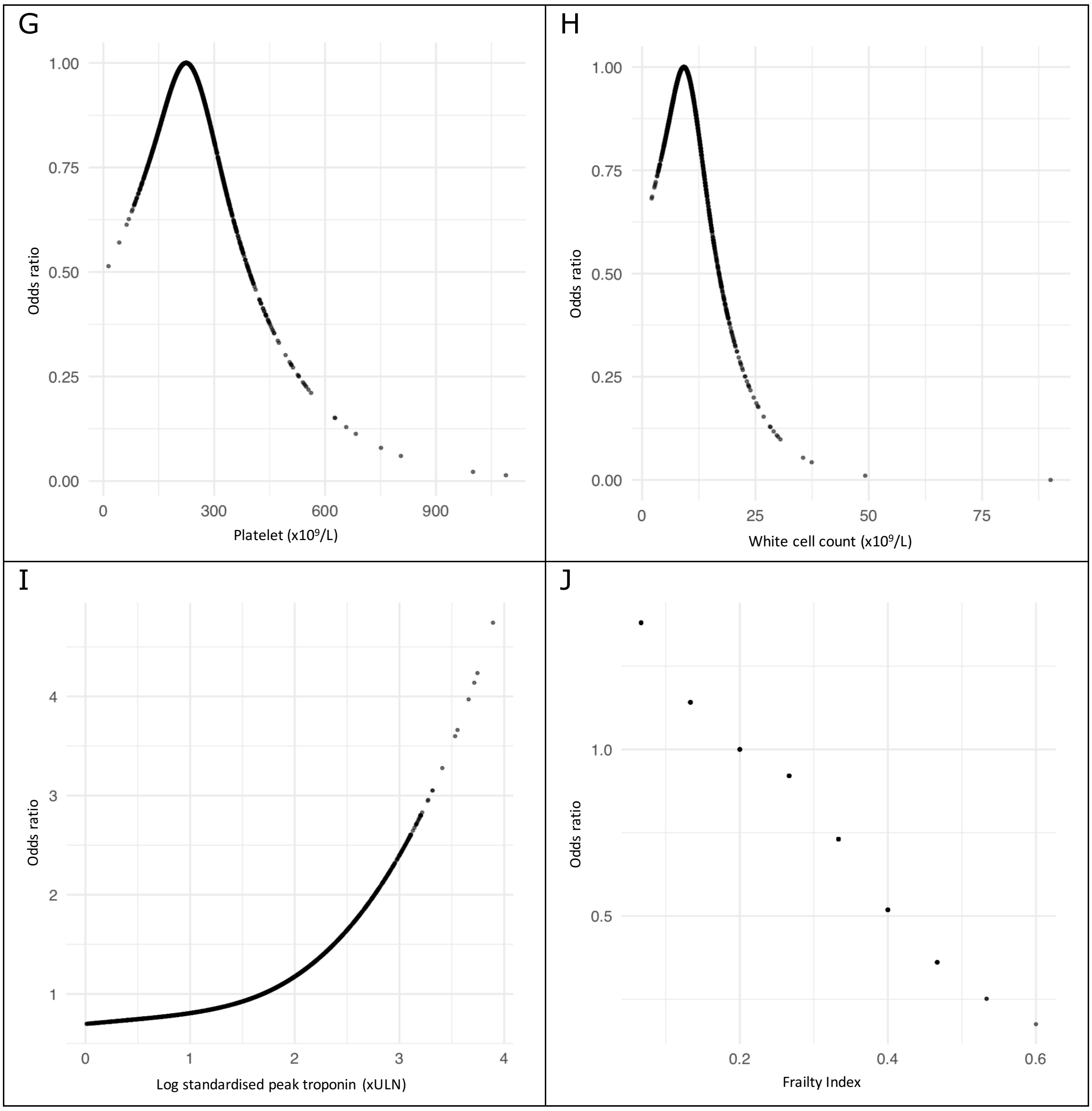

The figure shows the odds ratios of undergoing invasive management according to the following variables: (A) age, (B) C-reactive protein, (C) creatinine, (D) haemoglobin, (E) sodium, (F) potassium, (G) platelet count, (H) white blood cell count, (I) log-standardised peak troponin level and (J) the comorbidity domain of the Frailty Index. In each figure the median of the variable was chosen as the comparator value (odds ratio = 1).

Supplementary Figure S3. **Unadjusted Kaplan-Meier mortality curves displaying cumulative all-cause mortality and probability of admission for heart failure according to invasive and non-invasive management.**

A All-cause mortality

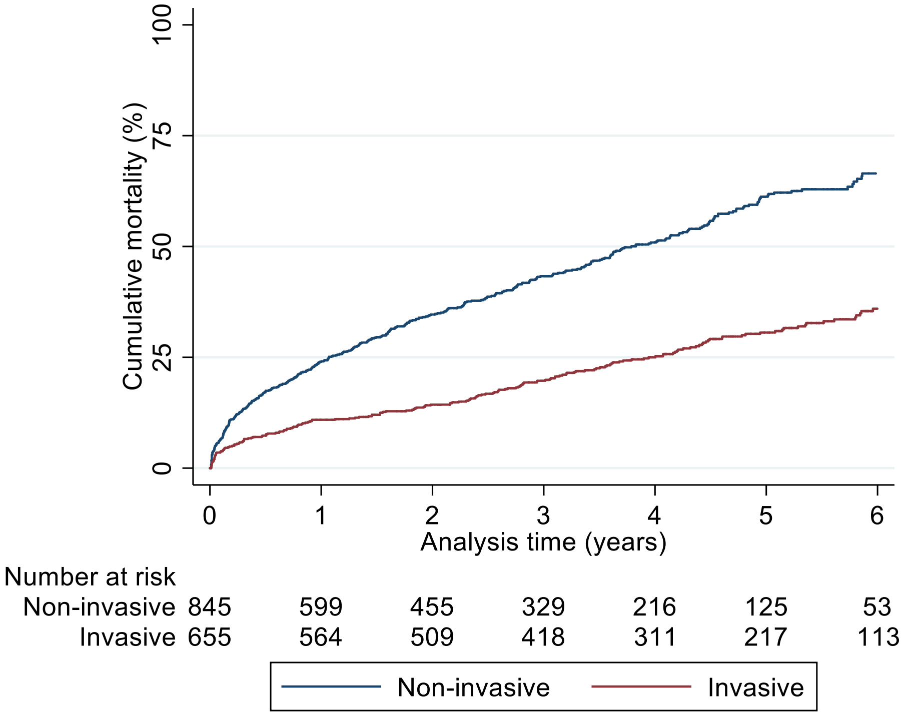

B Heart failure admission

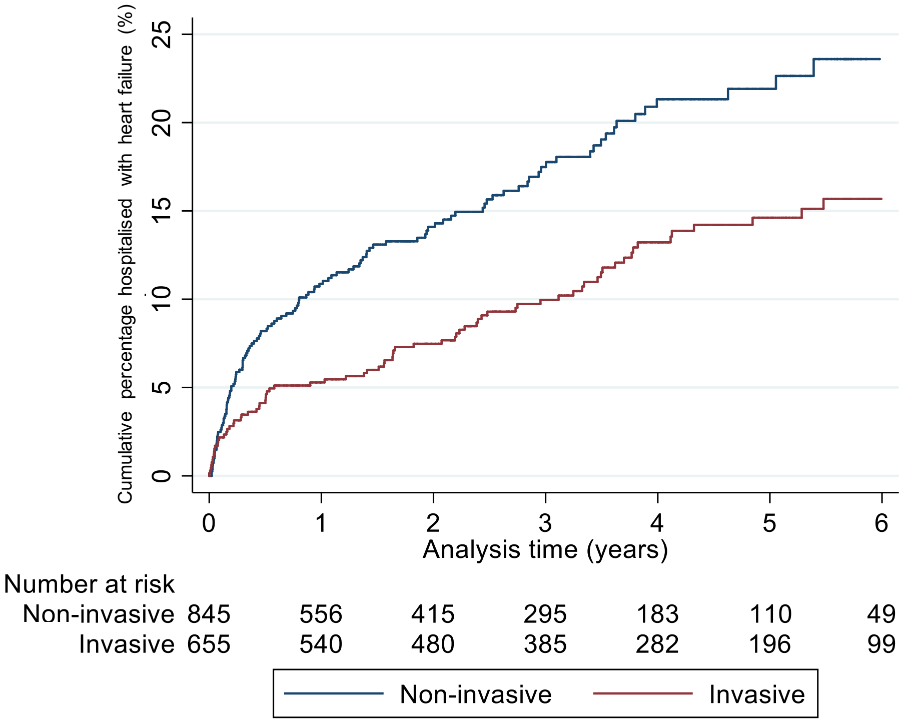

Patient deaths within 3 days of peak troponin were excluded.

Supplementary Figure S4. Probability density functions of the propensity score for the 101 patients treated invasively or non-invasively who died within 3 days of their peak troponin

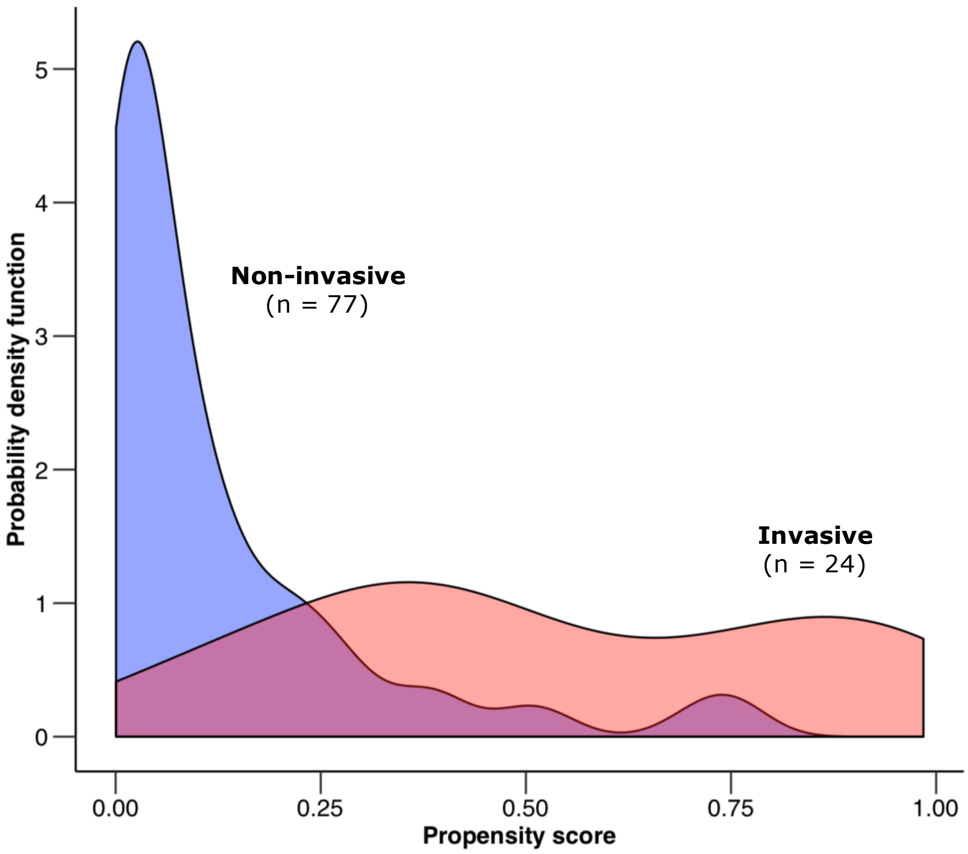

Supplementary Figure S5. **Estimated mortality hazard ratio for invasive versus non-invasive management according to age**

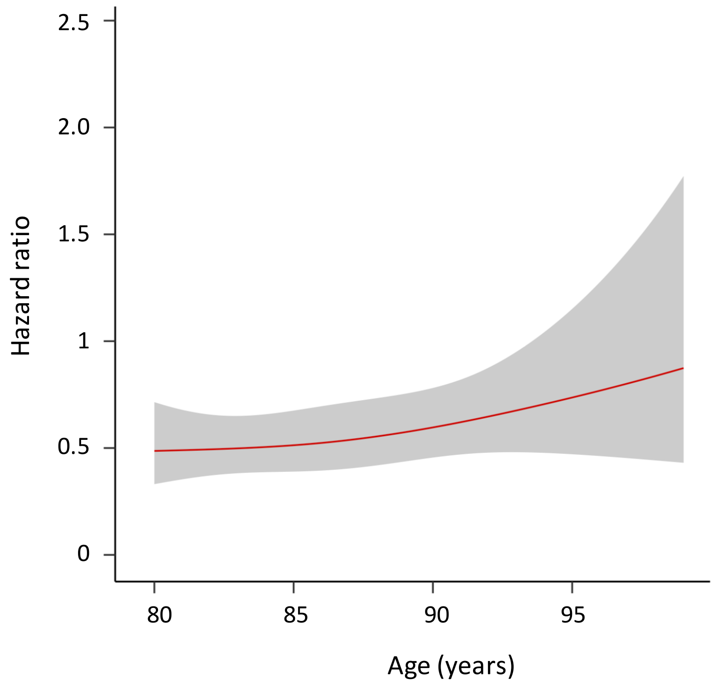

Restricted cubic spline model adjusted for propensity score, age, interhospital transfer, creatinine, haemoglobin, family history of ischaemic heart disease, hypercholesterolemia, hypertension, abdominal aortic aneurysm, angina, aortic stenosis, cardiogenic shock, heart failure, previous myocardial infarction, supraventricular tachycardia, ventricular fibrillation, ventricular tachycardia, acute renal failure, urinary tract infection, interstitial lung disease, obstructive lung disease, other lung disease, ischaemic stroke, Parkinson's disease, anxiety, gastric ulcer, metabolic disorder, bowel incontinence, dementia, history of falls, mild cognitive impairment (no dementia), need for mobility assistance, and speaking difficulty. Patient deaths within 3 days of peak troponin were excluded.

Supplementary Table S1. Variables considered and included (based on backwards stepwise selection with p-value threshold 0.2) in each model

	Included in propensity score model	Included in Cox model for mortality	Included in negative binomial model for heart failure admission	Included in Cox model for bleeding or haemorrhage admission	Included in Cox model for stroke admission	Included in Cox model for acute coronary syndrome admission	Included in Cox model for further invasive management admission
**Demographic characteristics**							
Age (years)	Yes	Yes	Yes			Yes	Yes
Hospital	Yes	Stratified[#]	Yes	Stratified[#]	Stratified[#]	Stratified[#]	Stratified[#]
Interhospital transfer	Yes	Yes	Yes				
Male sex	Yes		Yes		Yes		
**Haematology and biochemistry results**							
C-reactive protein (mg/L)	Yes					Yes	
Creatinine (μmol/L)		Yes	Yes		Yes		Yes
Haemoglobin (g/dL)	Yes	Yes			Yes		
Platelet count (x10^9/L)	Yes					Yes	Yes
Potassium (mmol/L)				Yes			
Sodium (mmol/L)			Yes		Yes	Yes	
Log standardised peak troponin (xULN)	Yes		Yes				
White cell count (x10^9/L)	Yes				Yes		
**Cardiovascular risk factors**							
Diabetes mellitus					Yes		
Family history of ischaemic heart disease	Yes	Yes		Yes		Yes	Yes
Hypercholesterolemia		Yes	Yes				
Hypertension	Yes	Yes					
Tobacco use	Yes		Yes			Yes	Yes
**Cardiovascular disease**							
Abdominal aortic aneurysm		Yes			Yes		
Angina	Yes	Yes					
Aortic stenosis	Yes	Yes	Yes				Yes
Atrial fibrillation	Yes					Yes	Yes
Cardiogenic shock	Yes	Yes					
Cardiac arrest			Yes				Yes
Complete heart block							Yes
Heart failure	Yes	Yes	Yes				
Peripheral vascular disease							
Previous myocardial infarction	Yes	Yes	Yes			Yes	Yes
Supraventricular tachycardia		Yes				Yes	
Ventricular fibrillation		Yes					
Ventricular tachycardia	Yes	Yes	Yes				
**Renal disease**							
Acute renal failure	Yes	Yes		Yes	Yes		
Chronic kidney disease (>stage 2)					Yes		
Urinary tract infection	Yes	Yes					
**Respiratory disease**							
Interstitial lung disease	Yes	Yes					

	Included in propensity score model	Included in Cox model for mortality	Included in negative binomial model for heart failure admission	Included in Cox model for bleeding or haemorrhage admission	Included in Cox model for stroke admission	Included in Cox model for acute coronary syndrome admission	Included in Cox model for further invasive management admission
Obstructive lung disease		Yes					
Other lung disease	Yes	Yes				Yes	
Pneumonia	Yes		Yes				
Pulmonary embolism					Yes		
Respiratory failure	Yes		Yes				Yes
**Neurological disease**							
Ischaemic stroke	Yes	Yes					
Parkinson’s disease		Yes					
Subdural haemorrhage							
**Psychiatric disease**							
Anxiety	Yes	Yes					
Alcohol abuse	Yes		Yes	Yes			
Bipolar disorder	Yes				Yes		
Delirium	Yes						
Depression							
Other psychiatric illness	Yes					Yes	
**Other comorbidities**							
Arthritis	Yes		Yes			Yes	
Constipation			Yes	Yes			
Fracture							
Gastric ulcer		Yes					
Haemorrhage	Yes						
Inflammatory disorder							
Malignancy	Yes		Yes	Yes	Yes		Yes
Metabolic disorder		Yes					
Sepsis			Yes	Yes			
**Frailty**							
Bowel incontinence	Yes	Yes	Yes			Yes	Yes
Comorbidity Domain of Frailty Index score			Yes		Yes	Yes	
Dementia	Yes	Yes	Yes				
History of falls	Yes	Yes		Yes			
Impaired hearing							
Impaired vision							Yes
Mild cognitive impairment, no dementia		Yes			Yes		
Need for assistance at home	Yes		Yes				
Need for mobility assistance	Yes	Yes		Yes			
Need for personal care assistance	Yes						
Reduced mobility	Yes				Yes		
Speaking difficulty	Yes	Yes					
Urinary catheterisation							
Urinary incontinence	Yes		Yes				
Weight loss	Yes						

*baseline hazard function stratified by centre of admission.

Supplementary Table S2. Characteristics of the 696 patients with NSTEMI who had a concurrent primary diagnosis with an acute illness

Primary diagnosis with acute illness	Number (%)
Pneumonia	149 (5.6)
Acute heart failure	107 (4.0)
Inflammatory disorder	98 (3.7)
Stroke	84 (3.1)
Fracture	47 (1.8)
Sepsis	43 (1.6)
Renal disease	35 (1.3)
Respiratory failure	24 (0.9)
Abdominal aortic aneurysm	23 (0.9)
Haemorrhage	21 (0.8)
Metabolic disorder	16 (0.6)
Atrial fibrillation	15 (0.6)
Complete heart block	13 (0.5)
Pulmonary embolism	9 (0.3)
Ventricular tachycardia / fibrillation	8 (0.3)
Supraventricular tachycardia	3 (0.1)
Cardiogenic shock	1 (0.04)

Supplementary Table S3. Characteristics (median [interquartile range] or number [%]) of the 1976 patients undergoing NSTEMI stratified by study subgroup.

	Study population (n=1500)	Died at <3 days (n=101)	Excluded: propensity score <10th percentile^{+} (n=188)	Excluded: propensity score >90th percentile^{+} (n=187)
**Demographic characteristics**				
Age (years)	86 (82, 89)	85 (83, 88)	91 (87, 95)	83 (81, 86)
Interhospital transfer	105 (7.0%)	7 (6.9%)	1 (0.5%)	93 (49.7%)
Male sex	808 (53.9%)	49 (48.5%)	57 (30.3%)	136 (72.7%)
**Haematology and biochemistry results**				
C-reactive protein (mg/L)	8.3 (3.0, 33.1)	27.7 (14.0, 74.9)	35.5 (7.2, 93.9)	5.0 (2.1, 14.7)
Creatinine (µmol/L)	94 (75, 125)	132 (96, 187)	97 (77, 133)	98 (78, 121)
Haemoglobin (g/dL)	12.3 (11.0, 13.6)	11.8 (10.1, 13.1)	11.9 (10.4, 13.4)	13.2 (12.1, 14.0)
Platelet count (x10^9/L)	223 (179, 273)	224 (189, 282)	227 (180, 315)	213 (185, 260)
Potassium (mmol/L)	4.3 (3.9, 4.6)	4.6 (4.2, 5.2)	4.4 (3.9, 4.8)	4.2 (3.8, 4.5)
Sodium (mmol/L)	138 (135, 140)	138 (135, 141)	138 (135, 140)	138 (135, 140)
Troponin (xULN)	47 (8, 187)	191 (38, 478)	23 (8, 85)	188 (45, 722)
White cell count (x10^9/L)	9.2 (7.1, 11.7)	12.5 (9.8, 16.6)	10.5 (7.9, 14.6)	9.2 (7.6, 11.5)
**Cardiovascular risk factors**				
Diabetes mellitus	373 (24.9%)	27 (26.7%)	30 (16.0%)	44 (23.5%)
Family history of ischaemic heart disease	163 (10.9%)	2 (2.0%)	1 (0.5%)	72 (38.5%)
Hypercholesterolemia	528 (35.2%)	20 (19.8%)	28 (14.9%)	65 (34.8%)
Hypertension	862 (57.5%)	44 (43.6%)	63 (33.5%)	116 (62.0%)
Tobacco use	389 (25.9%)	5 (5.0%)	7 (3.7%)	153 (81.8%)
**Cardiovascular disease**				
Abdominal aortic aneurysm	10 (0.7%)	1 (1.0%)	2 (1.1%)	0 (0.0%)
Angina		7 (6.9%)		
Aortic stenosis	91 (6.1%)	6 (5.9%)	19 (10.1%)	0 (0.0%)
Atrial fibrillation	260 (17.3%)	14 (13.9%)	44 (23.4%)	20 (10.7%)
Cardiogenic shock	13 (0.9%)	15 (14.9%)	3 (1.6%)	1 (0.5%)
Cardiac arrest	23 (1.5%)	15 (14.9%)	5 (2.7%)	3 (1.6%)
Complete heart block	23 (1.5%)	5 (5.0%)	2 (1.1%)	3 (1.6%)
Heart failure	315 (21.0%)	45 (44.6%)	79 (42.0%)	12 (6.4%)
Peripheral vascular disease	86 (5.7%)	9 (8.9%)	4 (2.1%)	16 (8.6%)
Previous myocardial infarction	973 (64.9%)	61 (60.4%)	46 (24.5%)	173 (92.5%)
Supraventricular tachycardia	4 (0.3%)	1 (1.0%)	0 (0.0%)	1 (0.5%)
Ventricular fibrillation	12 (0.8%)	1 (1.0%)	0 (0.0%)	3 (1.6%)
Ventricular tachycardia	17 (1.1%)	2 (2.0%)	0 (0.0%)	7 (3.7%)
**Renal disease**				
Acute renal failure	107 (7.1%)	21 (20.8%)	25 (13.3%)	5 (2.7%)
Chronic kidney disease (>stage 2)	118 (7.9%)	16 (15.8%)	25 (13.3%)	8 (4.3%)
Urinary tract infection	77 (5.1%)	5 (5.0%)	41 (21.8%)	1 (0.5%)
**Respiratory disease**				
Interstitial lung disease	33 (2.2%)	0 (0.0%)	11 (5.9%)	0 (0.0%)
Obstructive lung disease	201 (13.4%)	15 (14.9%)	32 (17.0%)	16 (8.6%)
Other lung disease	181 (12.1%)	16 (15.8%)	35 (18.6%)	5 (2.7%)
Pneumonia	113 (7.5%)	19 (18.8%)	51 (27.1%)	2 (1.1%)
Pulmonary embolism	5 (0.3%)	2 (2.0%)	1 (0.5%)	0 (0.0%)
Respiratory failure	34 (2.3%)	9 (8.9%)	12 (6.4%)	0 (0.0%)
**Neurological disease**				
Ischaemic stroke	23 (1.5%)	3 (3.0%)	10 (5.3%)	0 (0.0%)
Parkinson's disease	12 (0.8%)	2 (2.0%)	2 (1.1%)	0 (0.0%)

	Study population (n=1500)	Died at <3 days (n=101)	Excluded: propensity score <10th percentile+ (n=188)	Excluded: propensity score >90th percentile+ (n=187)
Subdural haemorrhage	3 (0.2%)	0 (0.0%)	1 (0.5%)	0 (0.0%)
**Psychiatric disease**				
Alcohol abuse	64 (4.3%)	13 (12.9%)	21 (11.2%)	2 (1.1%)
Anxiety	19 (1.3%)	1 (1.0%)	11 (5.9%)	0 (0.0%)
Bipolar disease	5 (0.3%)	19 (18.8%)	5 (2.7%)	0 (0.0%)
Delirium	18 (1.2%)	3 (3.0%)	16 (8.5%)	0 (0.0%)
Depression	27 (1.8%)	1 (1.0%)	6 (3.2%)	3 (1.6%)
Other psychiatric illness	150 (10.0%)	6 (5.9%)	30 (16.0%)	9 (4.8%)
**Other comorbidities**				
Arthritis	50 (3.3%)	15 (14.9%)	13 (6.9%)	3 (1.6%)
Constipation	37 (2.5%)	2 (2.0%)	17 (9.0%)	4 (2.1%)
Fracture	17 (1.1%)	4 (4.0%)	10 (5.3%)	1 (0.5%)
Gastric ulcer	6 (0.4%)	1 (1.0%)	3 (1.6%)	0 (0.0%)
Haemorrhage	54 (3.6%)	4 (4.0%)	10 (5.3%)	4 (2.1%)
Inflammatory disorder	171 (11.4%)	12 (11.9%)	34 (18.1%)	8 (4.3%)
Malignancy	124 (8.3%)	14 (13.9%)	25 (13.3%)	16 (8.6%)
Metabolic disorder	28 (1.9%)	3 (3.0%)	10 (5.3%)	2 (1.1%)
Sepsis	28 (1.9%)	4 (4.0%)	4 (2.1%)	0 (0.0%)
**Frailty**				
Bowel incontinence	47 (3.1%)	0 (0.0%)	47 (25.0%)	1 (0.5%)
Comorbidity Domain of Frailty Index score	0.20 (0.13, 0.27)	0.27 (0.20, 0.33)	0.27 (0.20, 0.33)	0.20 (0.13, 0.27)
Dementia	50 (3.3%)	8 (7.9%)	32 (17.0%)	0 (0.0%)
History of falls	79 (5.3%)	4 (4.0%)	59 (31.4%)	2 (1.1%)
Impaired hearing	28 (1.9%)	14 (13.9%)	17 (9.0%)	3 (1.6%)
Impaired vision	18 (1.2%)	0 (0.0%)	12 (6.4%)	3 (1.6%)
Mild cognitive impairment, no dementia	24 (1.6%)	13 (12.9%)	6 (3.2%)	1 (0.5%)
Need for assistance at home	170 (11.3%)	16 (15.8%)	49 (26.1%)	10 (5.4%)
Need for mobility assistance	125 (8.3%)	38 (37.6%)	50 (26.6%)	6 (3.2%)
Need for personal care assistance	28 (1.9%)	0 (0.0%)	18 (9.6%)	0 (0.0%)
Reduced mobility	35 (2.3%)	3 (3.0%)	32 (17.0%)	0 (0.0%)
Speaking difficulty	28 (1.9%)	0 (0.0%)	6 (3.2%)	0 (0.0%)
Urinary catheterisation	16 (1.1%)	0 (0.0%)	2 (1.1%)	1 (0.5%)
Urinary incontinence	32 (2.1%)	1 (1.0%)	17 (9.0%)	0 (0.0%)
Weight loss	30 (2.0%)	1 (1.0%)	13 (6.9%)	0 (0.0%)

+ Patients with propensity score <10th percentile were very likely to receive non-invasive intervention: those with propensity scores >90th percentile were very likely to receive invasive intervention

Supplementary Table S4. **Characteristics of 1875 patients with NSTEMI aged 80 or over who survived for 3 days after peak troponin, according to invasive or non-invasive management strategy.**

Disease/characteristic	Invasive management (n=836)	Non-invasive management (n=1039)	
**Numerical characteristics**			**Mean difference (95% CI)**
**Demographic characteristics**			
Age (years)	85.0 (4.1)	87.5 (5.2)	2.6 (2.1, 3.0)
**Haematology and biochemistry results**			
C-reactive protein (mg/L)	23.5 (42.4)	40.8 (61.4)	17.3 (12.4, 22.2)
Creatinine (μmol/L)	114.5 (94.5)	120 (89.4)	5.6 (-2.7, 14.0)
Haemoglobin (g/dL)	12.6 (1.8)	12.1 (2.0)	-0.5 (-0.7, -0.4)
Platelet count (x10^9/L)	228.2 (71.3)	240.6 (93.3)	12.4 (4.8, 20.1)
Potassium (mmol/L)	4.3 (0.6)	4.3 (0.6)	0.1 (0.0, 0.1)
Sodium (mmol/L)	137.0 (4.1)	137.2 (4.8)	0.2 (-0.3, 0.6)
Troponin (xULN)	313.0 (527.8)	171 (426)	-142 (-185, -98.5)
White cell count (x10^9/L)	9.6 (3.4)	10.4 (5.1)	0.9 (0.5, 1.3)
**Frailty**			
Comorbidity Domain of Frailty Index	0.21 (0.09)	0.23 (0.10)	0.02 (0.01, 0.03)
**Binary characteristics**			**Odds ratio (95% CI)**
**Demographic characteristics**			
Interhospital transfer	162 (19.4%)	37 (3.6%)	6.51 (4.50, 9.43)
Male (vs female)	522 (62.4%)	479 (46.1%)	1.94 (1.61, 2.34)
**Cardiovascular risk factors**			
Diabetes mellitus	213 (25.5%)	234 (22.5%)	1.18 (0.95, 1.46)
Family history of IHD	178 (21.3%)	58 (5.6%)	3.06 (2.07, 4.53)
Hypercholesterolaemia	336 (40.2%)	285 (27.4%)	1.78 (1.46, 2.16)
Hypertension	516 (61.7%)	525 (50.5%)	1.58 (1.31, 1.90)
Tobacco use	387 (46.3%)	162 (15.6%)	4.67 (3.76, 5.79)
**Cardiovascular disease**			
Abdominal aortic aneurysm	3 (0.4%)	9 (0.9%)	0.41 (0.11, 1.53)
Angina	162 (19.4%)	183 (17.6%)	1.12 (0.89, 1.42)
Aortic stenosis	34 (4.1%)	76 (7.3%)	0.54 (0.35, 0.81)
Atrial fibrillation	124 (14.8%)	200 (19.3%)	0.73 (0.57, 0.93)
Cardiogenic shock	7 (0.8%)	10 (1.0%)	0.87 (0.33, 2.29)
Cardiac arrest	13 (1.6%)	18 (1.7%)	0.90 (0.44, 1.84)
Complete heart block	12 (1.4%)	16 (1.5%)	0.93 (0.44, 1.98)
Heart failure	122 (14.6%)	284 (27.3%)	0.45 (0.36, 0.57)
Peripheral vascular disease	55 (6.6%)	51 (4.9%)	1.36 (0.92, 2.02)
Previous myocardial infarction	667 (79.8%)	525 (50.5%)	3.86 (3.14, 4.76)
Supraventricular tachycardia	2 (0.2%)	3 (0.3%)	0.83 (0.14, 4.97)
Ventricular fibrillation	8 (1.0%)	7 (0.7%)	1.42 (0.51, 3.94)
Ventricular tachycardia	15 (1.8%)	9 (0.9%)	2.09 (0.91, 4.80)
**Renal disease**			
Acute renal failure	48 (4.6%)	99 (9.5%)	0.45 (0.31, 0.66)
CKD (>stage 2)	56 (6.7%)	95 (9.1%)	0.71 (0.51, 1.01)
Urinary tract infection	18 (2.2%)	101 (9.7%)	0.20 (0.12, 0.34)
**Respiratory disease**			
Interstitial lung disease	5 (0.6%)	39 (3.8%)	0.15 (0.06, 0.39)
Obstructive lung disease	100 (12.0%)	149 (14.3%)	0.81 (0.62, 1.06)
Other lung disease	70 (8.4%)	151 (14.5%)	0.54 (0.40, 0.73)
Pneumonia	47 (4.4%)	129 (12.4%)	0.33 (0.22, 0.48)
Pulmonary embolism	3 (0.4%)	3 (0.3%)	1.24 (0.25, 6.18)
Respiratory failure	9 (1.1%)	37 (3.6%)	0.29 (0.14, 0.61)
**Neurological disease**			
Ischaemic stroke	6 (0.7%)	27 (2.6%)	0.09 (0.01, 0.72)
Parkinson's disease	5 (0.6%)	9 (0.9%)	0.69 (0.30, 2.06)
Subdural haemorrhage	2 (0.2%)	2 (0.2%)	1.24 (0.17, 8.85)
**Psychiatric disease**			
Alcohol abuse	22 (2.6%)	65 (6.3%)	0.40 (0.25, 0.66)
Anxiety	4 (0.5%)	26 (2.5%)	0.19 (0.07, 0.54)
Bipolar disease	1 (0.1%)	9 (0.9%)	0.14 (0.02, 1.08)
Delirium	3 (0.4%)	31 (3.0%)	0.12 (0.04, 0.38)

Disease/characteristic	Invasive management (n=836)	Non-invasive management (n=1039)	
Depression	12 (1.4%)	24 (2.3%)	0.62 (0.31, 1.24)
Other psychiatric illness	68 (8.1%)	121 (11.7%)	0.67 (0.49, 0.92)
**Other comorbidities**			
Arthritis	16 (1.9%)	50 (4.8%)	0.39 (0.22, 0.68)
Constipation	18 (2.2%)	40 (3.9%)	0.55 (0.31, 0.97)
Fracture	6 (0.7%)	22 (2.1%)	0.33 (0.13, 0.83)
Gastric ulcer	2 (0.2%)	7 (0.7%)	0.35 (0.07, 1.71)
Haemorrhage	27 (3.2%)	41 (4.0%)	0.81 (0.50, 1.33)
Inflammatory disorder	65 (7.8%)	148 (14.2%)	0.51 (0.37, 0.69)
Malignancy	59 (7.1%)	106 (10.2%)	0.67 (0.48, 0.93)
Metabolic disorder	7 (0.8%)	33 (3.2%)	0.26 (0.11, 0.58)
Sepsis	6 (0.7%)	26 (2.5%)	0.28 (0.12, 0.69)
**Frailty**			
Bowel incontinence	12 (1.4%)	83 (8.0%)	0.17 (0.09, 0.31)
Dementia	9 (1.1%)	73 (7.0%)	0.14 (0.07, 0.29)
History of falls	18 (2.2%)	122 (11.7%)	0.17 (0.10, 0.27)
Impaired hearing	9 (1.1%)	39 (3.8%)	0.28 (0.13, 0.58)
Impaired vision	6 (0.7%)	27 (2.6%)	0.27 (0.11, 0.66)
Mild cognitive impairment	6 (0.7%)	25 (2.4%)	0.29 (0.12, 0.72)
Need for assistance at home	67 (8.0%)	162 (15.6%)	0.47 (0.35, 0.64)
Need for mobility assistance	47 (5.6%)	134 (12.9%)	0.40 (0.28, 0.57)
Need for personal care assistance	6 (0.7%)	40 (3.9%)	0.18 (0.08, 0.43)
Reduced mobility	9 (1.1%)	58 (5.6%)	0.18 (0.09, 0.37)
Speaking difficulty	6 (0.7%)	28 (2.7%)	0.26 (0.11, 0.63)
Urinary catheterisation	6 (0.7%)	13 (1.3%)	0.57 (0.22, 1.51)
Urinary incontinence	14 (1.7%)	35 (3.4%)	0.49 (0.26, 0.91)
Weight loss	36 (3.5%)	7 (0.8%)	0.24 (0.10, 0.53)

Characteristics are tabulated as mean (standard deviation) for numerical variables and number (%) for binary variables. Comparisons between groups are unadjusted and are quantified as mean differences for numerical characteristics and odds ratios for binary characteristics.

Supplementary Table S5. Estimated effect of invasive versus non-invasive management on mortality in patients with NSTEMI aged 80 and over, according to length of follow-up. Deaths during the first three days were excluded.

Follow-up period	Number of deaths		Adjusted* hazard ratio (95% CI)
	Invasive management	Non-invasive management	
Up to 1 month	23	52	1.02 (0.61-1.71)
1-2.99 months	12	50	0.51 (0.27-0.98)
3-5.99 months	13	41	0.62 (0.33-1.18)
6-11.99 months	23	57	0.74 (0.45-1.22)
1-2.99 years	51	135	0.46 (0.32-0.65)
≥3 years‡	63	93	0.49 (0.34-0.69)
Full follow-up‡	185	428	0.56 (0.45-0.69)

*Model adjusted for propensity score, as well as age, interhospital transfer, creatinine, haemoglobin, family history of ischaemic heart disease, hypercholesterolemia, hypertension, abdominal aortic aneurysm, angina, aortic stenosis, cardiogenic shock, heart failure, previous myocardial infarction, supraventricular tachycardia, ventricular fibrillation, ventricular tachycardia, acute renal failure, urinary tract infection, interstitial lung disease, obstructive lung disease, other lung disease, ischaemic stroke, Parkinson’s disease, anxiety, gastric ulcer, metabolic disorder, bowel incontinence, dementia, history of falls, mild cognitive impairment (no dementia), need for mobility assistance, and speaking difficulty.

Patient deaths within 3 days of peak troponin were excluded. ‡ median follow-up: 3-years

Supplementary Table S6. **Number (%) of patients who received repeat invasive management (invasive group) or first invasive management (non-invasive group) according to time since peak troponin.**

	**Invasive management (n=655)**	**Non-invasive management (n=845)**
4-6 days	9 (1.4%)	62 (7.3%)
7-14 days	10 (1.5%)	39 (4.6%)
15-28 days	7 (1.1%)	19 (2.3%)
1-2.99 months	11 (1.7%)	14 (1.7%)
3-11.99 months	39 (6.0%)	15 (1.8%)
1-1.99 years	15 (2.3%)	9 (1.1%)
2-2.99 years	4 (0.6%)	2 (0.2%)
≥3 years	8 (1.2%)	1 (0.1%)
Total	103 (15.7%)	161 (19.1%)

Supplementary Table S7. **Adjusted hazard ratios for hospital admissions for bleeding, stroke, acute coronary syndrome, and further invasive management. All comparisons are of invasive with non-invasive management in patients with NSTEMI aged 80 and over**

Outcome: Hospital admission for…	N (%) invasive	N (%) non-invasive	Adjusted hazard ratio (95% CI)*	P-value
Heart failure+	76	127	0.67 (0.48, 0.93)	0.019
Bleeding	33 (5.0%)	40 (4.7%)	0.93 (0.52, 1.65)	0.801
Stroke	18 (2.8%)	18 (2.1%)	1.44 (0.61, 3.40)	0.404
Acute coronary syndrome	65 (9.9%)	79 (9.4%)	0.67 (0.45, 1.00)	0.048
Further invasive management	103 (15.7%)	161 (19.1%)	0.46 (0.34, 0.61)	<0.001

* Adjusted incidence rate ratio for heart failure admission. Each model is adjusted for different variables, selected using backwards stepwise selection with p value threshold 0.2. These variables are listed in Supplementary Table S1.

+ Numbers shown are of patients who were admitted at least once for heart failure. Of these, 32/76 (42.1%) and 45/127 (34.4%) were admitted more than once, in the invasive and non-invasive groups, respectively.

UK NIHR Health Informatics Collaborative SENIOR-NSTEMI Study
DATA ACQUISITION PLAN

Author: Amit Kaura
Approvers: Jamil Mayet / Ben Glampson

Introduction

This document outlines the specifications and procedures for the NIHR Health Informatics Collaborative (NIHR HIC) Cardiovascular research database and defines the processes for the collection of the NIHR HIC Cardiovascular research data and onward sharing with researchers. The central research database is held within Imperial College Healthcare NHS Trust (ICHNT).

Data for all patients receiving a troponin test are collected locally at the following Trusts and submitted pseudonymously to ICHNT:

- Imperial College Healthcare NHS Trust
- University College London Hospitals NHS Foundation Trust
- Oxford University Hospitals NHS Foundation Trust
- Kings College Hospital NHS Foundation Trust
- Guys and St Thomas' Hospital NHS Foundation Trust

This document covers the processes for local collection of data at all sites. The main database and systems are hosted at ICHNT as the lead organisation for the Cardiovascular Theme.

NIHR HIC Cardiovascular Database Definitions

Local data store

Each Trust will have a local store of NIHR HIC Cardiovascular data; this collects their information in an identifiable form from clinical systems. The data will then be de-identified within the local NHS Trust. These de-identified data will be passed to the central research database.

Research database

The research database will contain only de-identified information. This database combines the data from each site (including ICHNT) and will be used for the SENIOR-NSTEMI study. The database will contain some secondary patient identifiers (e.g. date of procedure, date of death) which will not be made directly available to researchers. A further anonymised view of the data will be prepared by ICHNT staff which will involve converting all dates to the number days since the first troponin test. This view of the data will contain only the variables necessary to complete the study rather than the full database.

Procedures for local data collection into local stores

Data will be collected automatically from primary clinical systems within each Trust. NHS staff will enter data into clinical systems during routine clinical care of patients, or data will be generated as a result of clinical tests. Data in the primary clinical systems will be processed in accordance with each Trusts clinical guidelines and subject to local quality and governance procedures. Data will be extracted automatically and validated processes for data extraction, transformation and loading (ETL) have been designed to import the data into the local secure data stores.

Access to Local data stores

Access to identified data will be limited to those justified and approved by the local information governance teams and will always be NHS staff in accordance with the duty of confidentiality required by law. Anonymisation procedures will be automated after implementation.

Local data stores will be the only areas that hold any patient identifiers. De-identification is completed at this stage prior to passing data into any research databases and datasets.

De-identification

The data will be pseudonymised locally within each Trust; anonymisation processes will be automated and set up by NHS staff in accordance with:

- advice from local information governance procedures
- the HIC Standard Operating Procedure (SOP) for data sharing and anonymisation
- the Clinical Data transfer policy

The data items summarised in Table 1 will be anonymised. Anonymisation will be approved locally by information governance teams before data are sent externally to ICHNT. De-identified data are then shared in accordance with the overarching data sharing agreement.

Demographic	Anonymisation
Local Identifier	Provided if NHS number is missing
NHS Number	Use local pseudonymisation algorithm (key to be retained at source) Rename field to subject ID
Family Name	To be removed – LOCAL use
Given Name	To be removed – LOCAL use
Date of Birth	YYYY
Date of Death	DD-MM-YYYY

Table 1. Anonymisation of data elements

Each site will hold two versions of the database, one identifiable and one with de-identified, pseudonymised data. The de-identified version is for use in research and shared with the central research database at ICHNT. The identifiable database is held so that if necessary

patients can be re-identified if it is of importance to re-contact the patient via their care team.

NHS numbers and hospital numbers are pseudonymised using locally approved procedures. Names are removed from the dataset and date of birth is transformed to year of birth. Date of death is shared, however is converted in to relevant survival rates on provision of data to researchers. Researchers will never see the full date of death or be able to calculate it from other information (all dates are provided as delta for first troponin). The provision of date of death has been agreed by each of the information governance offices at each of the sites in the following de-identification and anonymisation protocol for the SENIOR-NSTEMI Study:

- The exact date of death is required to evaluate mortality after diagnosis.
- Patients presenting with acute coronary syndromes have a high frequency of cardiac events and a high short-term mortality rate. An accurate measure of death is therefore required to fully evaluate this.
- To redact the date of death to year only would misrepresent the survival of these patients, particularly for those who survive for less than one year. The clinical leads at our BRCs have underlined the importance of having the date in full for the SENIOR-NSTEMI study.

Data validity and quality

Prior to pseudonymisation within the clinical systems, NHS number, Date of Birth and patient names will be automatically checked to remove duplication of patients.
Samples of data will be clinically validated by members of the clinical team to ensure that the transformation process is correct and data are attributed to the correct patients prior to pseudonymisation. After clinical validation is complete, the data will be transformed to a standardised XML format and validated (Figure 1).

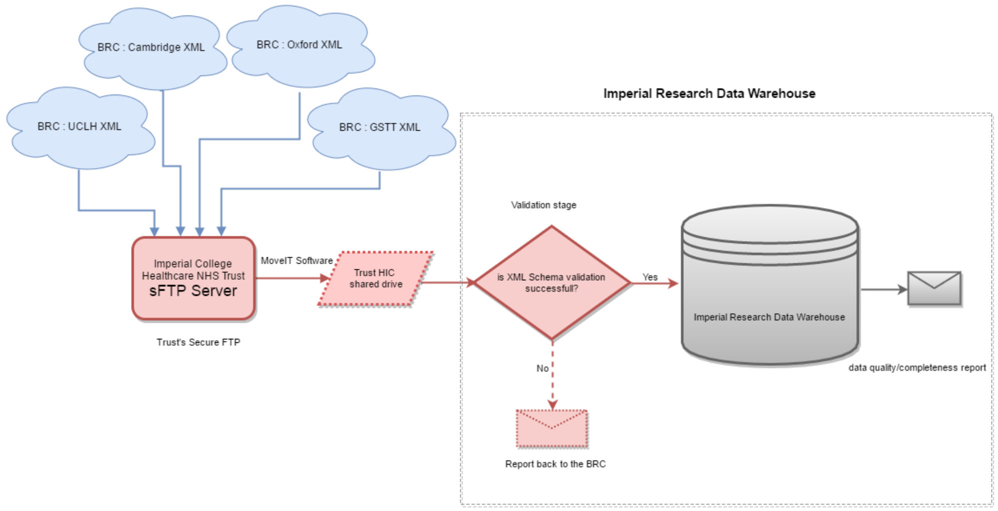

Figure 1. Data import and validation process.

Data sharing between Trusts

Data are shared in accordance with the SOP for data sharing. Data are encrypted in transit via sFTP on the N3 network (Figure 1). The sFTP is set up and hosted by ICHNT.

Procedures for secure research database

Data validity and quality

ICHNT validate the data to ensure that the structure, data items, units and data types are in accordance with the standardised data model. Data will be rejected if validation fails. On rejection, the files will be archived and the data manager will contact the data provider to review the submission and resend once corrected.
Once imported into the secure research database, data are subject to clinical validation by Cardiovascular clinical experts; these validations will be completed by the clinical researchers using de-identified data. Data will be reviewed for data completeness, spread and actual data point values. If data appears invalid it will be rejected.

Data quality reports will be generated and provided to the research team and local data provider after each submission. These will be reviewed after each submission to ensure all areas are populated.

Research Database software

The database is built using Microsoft SQL server 2014, Microsoft's principle database management system software. The installation of the software was carried out by certified technical consultants and tested by the Trust ICT team and data warehouse team in accordance with Trust ICT procedures and policies.

Database management

The database is fully backed up on a daily basis. The back-ups are standardised for all Trust databases within the Trust data warehouse. Backups can be restored at any point by warehouse staff. Data are secondary copies from clinical systems; at any point the participating Trusts can re-extract the data from primary sources.
Each site will submit data on a quarterly basis to the database. Data integrity checks will be completed to ensure correct structure is maintained and duplicates are not present.

Once entered on the system, data will not be changed. All access will be 'read only' except via exception, approved by the research Informatics Programme Manager and Clinical Leads group.

The database will be managed by the data manager (Ben Glampson) and developer (Abdul Mulla). Any database changes will be controlled by the research informatics Programme manager (Ben Glampson), and sanctioned by the NIHR HIC Cardiovascular scientific steering committee (Chaired by Jamil Mayet). All staff are substantive NHS employees at ICHNT.

Data extracts taken for research will be stored within the data warehouse and retained for the period specified in the data request. All information pertinent to the request will be retained and tracked by the data manager.

Data access for research

SENIOR-NSTEMI STUDY dataset

All analyses for the SENIOR-NSTEMI STUDY will be completed on fully de-identified data. This includes further de-identification to remove dates.

A designated clinical researcher (Amit) will freeze a copy of the database on 1st April 2017, so a static dataset can be used for analysis. This will be retained separately from the live database to allow reproducible analyses.

The study dataset will comprise all patients who had a troponin measured at each of the five academic centres between 2010 (2008 for University College Hospital) and 2017.

Dates will be converted to delta dates, with date zero being the date of the first troponin test. All further dates are provided as number of days from date zero. Age will be provided in years, at the time of the first troponin test.

Date of death will be converted to the number of days since date zero. All patients will be retrospectively followed up, using routinely collected data on the NHS Spine Application, Summary Care Record, until death or censoring on 1st April 2017.

Data elements

The database model will include 156 data points, grouped into demographics, emergency department attendance and inpatient episodes, biochemistry, diagnosis, angiography, revascularization, echocardiography and mortality. Diagnostic data will be based on International Statistical Classification of Diseases and Related Health Problems (ICD) discharge codes.
